# Genome-Wide Identification, Characterization, and Expression Profiling of the Legume *BZR* Transcription Factor Gene Family

**DOI:** 10.3389/fpls.2018.01332

**Published:** 2018-09-19

**Authors:** Yueying Li, Liangliang He, Jing Li, Jianghua Chen, Changning Liu

**Affiliations:** Key Laboratory of Tropical Plant Resources and Sustainable Use, Xishuangbanna Tropical Botanical Garden, Chinese Academy of Sciences, Mengla County, China

**Keywords:** genome-wide analysis, expression profiling, legume, BZR transcription factor, abiotic stresses

## Abstract

The BRASSINAZOLE-RESISTANT (BZR) family of transcription factors (TFs) are positive regulators in the biosynthesis of brassinosteroids. The latter is a class of steroid hormones that affect a variety of developmental and physiological processes in plants. BZR TFs play essential roles in the regulation of plant growth and development, including multiple stress-resistance functions. However, the evolutionary history and individual expression patterns of the legume *BZR* genes has not been determined. In this study, we performed a genome-wide investigation of the *BZR* gene family in seven legume species. In total, 52 *BZR* genes were identified and characterized. By analyzing their phylogeny, we divided these *BZR* genes into five groups by comparison with orthologs/paralogs in *Arabidopsis thaliana*. The intron/exon structural patterns and conserved protein motifs of each gene were analyzed and showed high group-specificities. Legume *BZR* genes were unevenly distributed among their corresponding genomes. Genome and gene sequence comparisons revealed that gene expansion of the BZR TF family in legumes mainly resulted from segmental duplications and that this family has undergone purifying selection. Synteny analysis showed that *BZR* genes tended to localize within syntenic blocks conserved across legume genomes. The expression patterns of *BZR* genes among various legume vegetative tissues and in response to different abiotic stresses were analyzed using a combination of public transcriptome data and quantitative PCR. The patterns indicated that many *BZR* genes regulate legume organ development and differentiation, and significantly respond to drought and salt stresses. This study may provide valuable information for understanding the evolution of *BZR* gene structure and expression, and lays a foundation for future functional analysis of the legume *BZR* genes by species and by gene.

## Introduction

The brassinosteroids (BRs) form a class of plant steroid hormones that play fundamental roles in a variety of developmental and physiological processes, including organ elongation, boundary formation as well as photomorphogenesis (Clouse and Sasse, 1998; Bajguz and Hayat, 2009). The hormones are widely distributed across the plant kingdom and produced within various plant organs such as root, shoot, leaf, and flower (Li and Chory, 1999; Fujioka and Yokota, 2003). Unlike animal steroid hormones that can directly regulate expression of target genes by binding to nuclear receptors, BRs function through a membrane-localized receptor, BRASSINOSTEROID-INSENSITIVE 1 (BRI1), and its signal transduction cascade targeting the regulation of the *BRASSINAZOLE-RESISTANT* (*BZR*) transcription factor (TF) gene family (Kim et al., 2009; Kim and Wang, 2010), which in turn controls the expression of 100s of target genes.

The *BZR* gene family encodes a class of novel plant-specific TFs that can directly mediate and regulate the BR signaling pathway by responding to BR binding and echoing a feedback signal (Li and Deng, 2005). Being positive regulators of BR signal transduction, BZR TFs have been proven to play essential roles in regulation of plant growth and development, and to be involved in multiple stress-response pathways (Wang et al., 2002; Yin et al., 2002; He et al., 2005). In the case of *Arabidopsis thaliana*, in which *BZR* genes have been well-studied, *BZR1* and *BRI1-EMS-SUPPRESSOR 1* (*BES1*) are the primary members of the *BZR* gene family, with at least four other homologs designated as *BES1/BZR1 Homolog1* (*BEH1*) to *BEH4* (Yin et al., 2005; Sun et al., 2010; Yu et al., 2011). Both *BZR1* and *BES1* have a basic helix-loop-helix (bHLH) DNA binding motif in the N-terminal domain that is highly conserved across the whole family, whereas their functions have diverged (Yin et al., 2005). *BZR1* binds to a BR-Response Element (CGTGT/CG motif) to suppress the expression of BR-biosynthetic genes (Wang et al., 2002), while *BES1* binds to an E box (CANNTG sequence) to activate BR-induced gene expression (Yin et al., 2002). In addition to a highly conserved N-terminal domain, the BZR proteins commonly possess 22–24 putative phosphorylation sites for members of the GLYCOGEN SYNTHASE KINASE-3 (GSK3) family in the central region, and some proteins also contain a putative PEST motif involved in protein degradation (Yin et al., 2005). Previous research has suggested that BZR TFs could regulate additional TFs so as to control a large number of downstream genes, and thus may perform a wider range of functions than initially thought (Sun et al., 2010; Yu et al., 2011). Therefore, identifying and characterizing new *BZR* genes from diverse plant species is a promising approach to obtain fresh insights into this highly conserved family.

Legumes (Fabaceae) are widely cultivated on about 12% of Earth’s arable land and account for about 27% of the world’s primary crop production (Graham and Vance, 2003). They can fix atmospheric nitrogen through association with symbiotic nitrogen-fixing bacteria in root nodules, and serve as an important source of nitrogen in crop rotation systems (Giller and Merckx, 2003). Legumes are likewise increasingly recognized as a source of valuable secondary metabolites, which serve as defense compounds against herbivores and microbes, as well as signal compounds to attract pollinating and fruit-dispersing animals (Dixon and Sumner, 2003). As some of the most important crops worldwide for both humans and domestic animals, further efforts should be made for mining and characterizing genes related to legume growth, development, and stress responses (Dixon and Sumner, 2003; Graham and Vance, 2003). The *BZR* genes encode important TFs that function in the regulation of plant growth and development and the BZR-mediated abiotic stress response and have been identified and characterized in detail in only a few plant species, including *A. thaliana*, *Brassica rapa*, and *Zea mays* (Yin et al., 2005; Saha et al., 2015; Manoli et al., 2018). Some predictions in public databases (such as iTAK and PlantTFDB) resulting from genome-scale automatic annotation pipelines have resulted in identification of new *BZR* genes (Zheng et al., 2016; Jin et al., 2017); however, the genome-wide identification, characterization, and expression profile analysis of *BZR* gene evolution and function in legumes has yet to be completed.

In the present study, we performed a genome-wide investigation of the *BZR* gene family in seven legume species occupying different clades of the Papilionoideae sub-family (Wojciechowski et al., 2004). In total, 52 *BZR* genes containing 56 *BZR* transcription sequences were identified using both local BLAST and hidden Markov model (HMM) based searches. By analyzing their molecular phylogeny using the Neighbor-Joining method implemented in MEGA6 and the maximum likelihood method in the PhyML 3.0, we divided all these *BZR* genes along with those of *A. thaliana* into five well-defined groups based on total sequence similarity, which were further validated by our subsequent analysis of gene intron/exon structural patterns and conserved sequence motifs. With intensive genome comparison, it was discovered that legume *BZR* genes vary in gene number and location and that the expansion of the *BZR* gene family in legumes mainly resulted from segmental duplication. Meanwhile, the *K*a/*K*s ratio (Zeng et al., 1998) of the duplicated *BZR* genes in seven species showed that this family had undergone purifying selection. Synteny relationships of these legume *BZR* genes were also explored, which showed *BZR* genes tended to localize within syntenic blocks conserved across legume genomes. Based on public transcriptome data and quantitative PCR expression analysis, we revealed the expression patterns of these *BZR* genes among various legume vegetative tissues and in response to different abiotic stresses, and found that many of them are involved in legume organ development and differentiation, and significantly responsive to drought and salt stresses. Our work provides valuable information for understanding the evolution of legume *BZR* genes, and lays a foundation for future functional analysis of *BZR* genes in legume growth and development and the BZR-mediated abiotic stress response.

## Materials and Methods

### Dataset

The genomic and proteomic sequences of seven legume species were downloaded from their respective databases: *Cajanus cajan* (pigeon pea, v-5.0^1^) (Varshney et al., 2011), *Cicer arietinum* (chickpea, CGAP_v1.0^2^) (Jain et al., 2013), *Glycine max* (soybean, Gmax_189^3^) (Schmutz et al., 2010), *Lotus japonicus* (build 2.5^4^) (Sato et al., 2008), *Medicago truncatula* (Mtruncatula_198^5^) (Young et al., 2011), *Phaseolus vulgaris* (common bean, Pvulgaris_218^6^) (Schmutz et al., 2014), and *Vigna radiata* (mung bean, Vr1.0^7^) (Dash et al., 2016).

### *BZR* Gene Identification and Characterization

To identify all the possible *BZR* genes in the seven legume genomes, both local BLAST and HMM searches (Stanke and Waack, 2003) were performed. The BZR protein sequences from *Arabidopsis* (TAIR 9.0 release) were taken as query to search for potential candidates against different leguminous proteomes via BLASTP (e-value < = 1e-10) (Altschul et al., 1990). In addition, protein sequences of the seven legumes were searched against HMM profiles of the BZR domain (PF05687) via HMMER (e-value < = 1) (Finn et al., 2015). The non-redundant protein sequences obtained from the above two approaches were further tested for the presence of a BZR domain by using SMART (Schultz et al., 1998) and NCBI Conserved Domains (Marchler-Bauer et al., 2015). For all identified BZR proteins, prediction of molecular weight, and isoelectric point was carried out using the ExPASy Proteomics Server (Artimo et al., 2012). The subcellular localizations of legume BZR proteins were predicted using ProtComp 9.0^8^. To identify the putative binding sites of possible upstream regulators of each legume *BZR* gene, the core promoter region (from -500 to +100 bp relative to a transcriptional start site) was analyzed using PlantRegMap (Jin et al., 2017). The functional annotation of each legume BZR protein was extracted from the RefSeq database (O’Leary et al., 2016).

### Phylogenetic Analysis

Multiple sequence alignments were performed on the legume BZR protein sequences using ClustalX (v2.1) with default parameters (Thompson et al., 2002). Gblocks (Castresana, 2000) was used to obtain the conserved sequences. A neighbor-joining tree was reconstructed using the MEGA program (v6.0) (Tamura et al., 2013) and bootstrapping was performed with 1000 replications. The maximum likelihood (ML) tree was generated with the program PhyML 3.0^9^ employing the JTT substitution model and the NNI heuristics. ML bootstrap support was calculated from 100 bootstrap replicates (Guindon et al., 2010).

### Gene Structure of BZR Proteins

Positional information for both the protein sequences and the corresponding coding sequences was loaded into the Gene Structure Display Server (GSDS v2.0) (Hu et al., 2015) to obtain information on intron/exon structure. The coordinates of the BZR domain in each protein were recalculated into the coordinates in the corresponding gene sequence and featured in gene structure.

### Detection of Additional Conserved Motifs

To identify additional conserved motifs outside the BZR domain of legume BZR proteins, we used MEME v4.11.2 (Bailey et al., 2009). The limits on maximum width, minimum width, and maximum number of motifs were specified as 6, 150, and 10, respectively. The motifs were numbered serially according to their order in MEME. Those motifs common to genes in one of the five similarity groups were designated as the group-specific signatures.

### Chromosomal Localization

According to the chromosomal positions of genes, we drew a map of the distribution of *BZR* genes throughout the seven legume genomes using MapInspect software^10^. The *BZR* gene pairs resulting from segmental or tandem duplication were linked by lines and marked in cyan, respectively.

### Detection of Duplicated Genes and Estimation of Synonymous (*K*s) and Non-synonymous (*K*a) Substitutions per Site and Their Ratio

Duplicated gene pairs derived from segmental or tandem duplication were identified in legume genomes based on the method described in the Plant Genome Duplication Database (Lee et al., 2013). An all-against-all BLASTP comparison (e-value < = 1e-20) provided the gene pairs for syntenic clustering determined by MCScan (e-value < = 1e-20) (Tang et al., 2008). Tandem duplication arrays were identified using BLASTP with a threshold of e-value < 1e-20, and one unrelated gene among cluster members was tolerated, as described for *A. thaliana* (The Arabidopsis Genome Initiative, 2000). Pairs from segmental and tandem duplications were used to estimate *K*a, *K*s, and their ratio. Coding sequences from segmentally and tandemly duplicated *BZR* gene pairs were aligned by PRANK (Loytynoja and Goldman, 2010) and trimmed by Gblocks. The software KaKs_Calculator (Wang et al., 2010) was then used to compute *K*a and *K*s values for each pair following the YN model (Yang and Nielsen, 2000).

### Synteny Relationships of *BZR* Genes

To compare the *BZR* genes in the seven legume species, as well as the non-legume model plant *Arabidopsis*, BLASTP searches between each pair were conducted using the predicted proteomes of all eight species (e-value ≤ 1e-10). The synteny blocks were then calculated by MCScanX (Wang et al., 2012). Proteins with unknown chromosomal loci were not used in the analysis. Ideograms were created using Circos (Krzywinski et al., 2009).

### Identification of Legume *BZR* Orthologs

Identification of putative orthologs of the *BZR* genes in the selected species was carried out as previously described (Blanc and Wolfe, 2004). Briefly, each sequence from one species was searched against all sequences from the other species using BLASTN and the same procedure was done conversely. Two sequences were defined as orthologs if each of them was the best match to the other and if the sequences were aligned over 300 bp or more.

### Expression Analysis of Legume *BZR* Genes

The original expression data for *BZR* genes in common bean, soybean, lotus, and *Medicago* were obtained from PvGEA^11^ (O’Rourke et al., 2014), Soyseq^12^ (Severin et al., 2010), LjGEA^13^ (Verdier et al., 2013), and MtGEA^14^ (Benedito et al., 2008), respectively. For visualization, data were log transformed and min–max normalized within each species according to the formula Y = (X_i_–X_min_)/(X_max_–X_min_) (Sriram et al., 2007), where X_i_ is each expression value, and X_max_ and X_min_ correspond to the maximum and minimum expression values in each matrix. For microarray expression data, the corresponding relationships between microarray probes and legume *BZR* genes were built using BLAST (best hit under 1e-10). The *pheatmap* package (version 1.0.08) was used to make a heatmap in R as previously reported (Wang et al., 2014)^15^.

### Collection and Preparation of Plant Material

For quantitative PCR expression analysis, soybean seeds [*G. max* L. cv. Williams 82] were soil-grown in greenhouses under the following controlled conditions: 24°C day/20°C night temperature; 16-h day/8-h night photoperiod; 30 to 50% relative humidity, and 150 μE/m^2^/s light intensity. The seedlings were randomly divided into three groups for drought treatment. Drought stress was applied by stop watering at 14 days after planting and drought stress period lasted for 14 days. The roots of treated samples were collected at 0, 2, 4, 7, 10, and 14 days after the beginning of the treatment, and processed for analysis of the expression patterns of different BZR TFs. Each tray was replicated three times, and three independent samples were collected from every tray.

### Quantitative PCR Expression Analysis

Real-time quantitative PCR was performed using 1 μL cDNA in a 10-μL reaction volume employing SYBR^®^ Premix Ex Taq^TM^ II (TAKARA, DRR081A) using the following gene-specific primer pairs: GmBZR2F (5′-CTGCTCCTCCTTCGCCTACC-3′) and GmBZR2R (5′-TCCATTCCAACCTCGTGTATTCTC-3′); GmBZR3F (5′-AGTCAGCAGCACAAGCACAAC-3′) and GmBZR3R (5′-AGCAGGCGTCTTCCCACTTC-3′); GmBZR8F (5′-GCGAGTGAGATTGGAGGAACAG-3′), and GmBZR8R (5′-GTATGACGAGGATTGTGGACTTGG-3′). A soybean actin gene Glyma.04G215900 was used as an internal control with primer pairs of GmACTIN-QRT-F (5′-ACTGGAATGGTGAAGGCAGG-3′) and GmACTIN-QRT-R (5′-CATTGTAAAATGTGTGATGCCAG-3′). The conditions for real-time PCR were as follows: 1 min at 95°C, followed by 40 cycles of 95°C for 15 s and 60°C for 30 s. The fluorescence was measured following the last step of each cycle, and three replications were used per sample. Amplification detection and data analysis were conducted using Graphpad prism5 (GraphPad Software, Inc., La Jolla, CA, United States).

## Results

### Identification and Characterization of *BZR* Genes in Seven Legume Genomes

Studies of the *BZR* gene family are mainly concentrated in the model plant *A. thaliana*, thus there were relatively few examples of *BZR* gene identification, characterization, and expression profiling analysis in other plant species, except those done as part of genome-scale predictions based on automatic annotation pipelines (**Table 1**). In this study, we performed a genome-wide investigation of the *BZR* gene family in seven legume species coming from different clades of the sub-family Papillionoideae, including one species from the Robinioids clade (*L. japonicus*), two from the Galegoid clade (*Cicer arietinum*, chickpea; *M. truncatula*), and four from the Phaseoloid clade (*Cajanus cajan*, pigeon pea; *G. max*, soybean; *P. vulgaris*, common bean; *V. radiata*, mung bean).

**Table 1:** *BZR* family members of legume and non-legume species.

Plant species	Common name	Number of genes	Representative genome size (Mb)	Reference
**Non-legume species**				
*Arabidopsis thaliana*	Thale cress	6	120	He et al., 2005; Kim and Wang, 2010
*Brassica rapa*	Bird rape	15	284	Saha et al., 2015
*Zea mays*	Maize	11	2135	Manoli et al., 2018
**Legume species**				
*Medicago truncatula* (Galegoid clade)	Barrel medic	7	413	This study
*Cicer arietinum (*Galegoid clade)	Chickpea	6	531	This study
*Glycine max* (Phaseoloid clade)	Soybean	16	979	This study
*Cajanus cajan* (Phaseoloid clade)	Pigeon pea	6	593	This study
*Phaseolus vulgaris* (Phaseoloid clade)	Common bean	7	521	This study
*Vigna radiata* (Phaseoloid clade)	Mung bean	5	464	This study
*Lotus japonicus* (Robinioids clade)	Birdsfoot trefoil	5	394	This study

To extensively identify *BZR* genes in each legume species, we used a whole-genome scan to identify genes that encode proteins containing the BZR DNA-binding domain by both BLASTP and HMM profile searches. To verify the reliability of our results, we further checked for the presence of the BZR domain using SMART and NCBI Conserved Domains analysis, and removed some candidates whose protein sequences had partial defects in the N-terminal region of the typical BZR domain (**Supplementary Table S1**). We identified 52 candidate *BZR* genes in total represented by 56 transcripts in seven legume species. For each legume species, the number of *BZR* genes generally varied from 5 to 7, including five in lotus, six in chickpea, seven in *Medicago*, seven in common bean, five in mung bean, and six in pigeon pea, with the one exception that soybean had 16 *BZR* genes (**Table 1**). This is likely because soybean possesses a partially diploidized tetraploid genome that might have undergone several duplications (Schmutz et al., 2010).

Depending upon their map coordinates on chromosomes/scaffolds, we designated each BZR protein uniquely, and used each species’ abbreviation as the prefix. Next, we systematically evaluated the basic properties of these BZR proteins, including the predicted protein length, domain position, molecular weight, isoelectric point, sub-cellular localization, and functional annotation (**Supplementary Table S2**). The predicted length of these BZR protein sequences displayed a bimodal distribution that peaks at 92 aa and 1296 aa with median length of 323 aa; for most of them (50 out of 56 proteins, ∼90%), the length centered on two ranges: (i) 240–340 amino acid residues for 35 BZR proteins and (ii) 650–710 amino acid residues for 15 BZR proteins. Correspondingly, the molecular masses were mainly distributed from 26.76 to 35.64 kDa and 73.71 to 79.30 kDa, with an median of 34.9 kDa. The predicted isoelectric point of BZR proteins varied from 5.07 to 9.87. The predicted subcellular localizations of legume BZR proteins indicated their presence in diverse organelles. Also, in accordance with their possible roles as TFs, half of these legume BZR proteins were determined to have nuclear localization. The functional annotation revealed that most of our identified genes (37 *BZR* genes) were annotated as BR signaling positive regulator-related proteins. However, interestingly, the remaining 15 genes were annotated as beta-amylase-(BAM) like proteins, in spite of the common BZR domain, indicating other functional domains apart from the BZR domain exist in these genes.

### Phylogenetic Analysis and Classification of Legume *BZR* Genes

**FIGURE 1:**
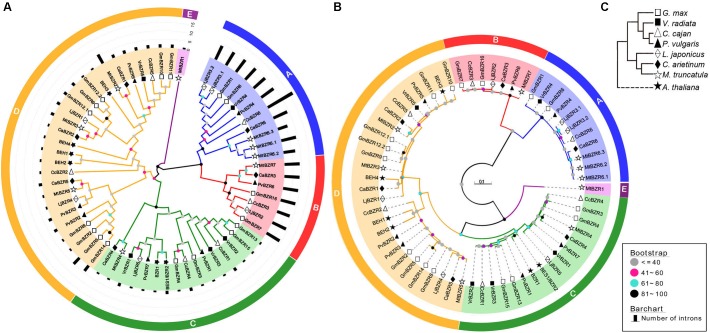
Neighbor-joining and maximum-likelihood phylogenetic analysis of legume and *Arabidopsis BZR* genes. **(A)** The neighbor-joining tree was created using MEGA6.0 with 1000 bootstrap replicates. Genes on branch ends from different species are denoted by different scatters. The legume BZR proteins were grouped into five distinct clades (A–E), which are indicated by colored branches. Intron number of *BZR* genes are denoted by black bars. **(B)** The maximum-likelihood tree was created using PhyML 3.0 (JTT +G+I+F) with 100 bootstrap replicates. The bootstrap values expressed as percentages in the key in A and B. **(C)** The hypothesized species relationships of these species. Different scatters indicate different species.

To explore phylogenetic relationships among BZR proteins in different legume species, a Neighbor-Joining phylogenetic tree was reconstructed including 56 legume and six *Arabidopsis* BZR proteins (**Figure 1A**). This analysis divided all BZR proteins into five major groups, named A, B, C, D, and E. The A, B, C, and E groups were supported by a bootstrap value of >80% that they are considered to be evidence for distinct phylogenetic lineages, while the nodes with a bootstrap value of below 60% were integrated into group D, in which there was great variation between members. This phylogenetic relationships was further supported by the maximum likelihood analysis that all groups were supported by a bootstrap value of >60% (**Figure 1B**). The hypothesized phylogenetic species relationships of the seven legumes and *A. thaliana* were shown in **Figure 1C**. It was shown that proteins with the same or similar domain organization are prone to cluster together (**Supplementary Data Sheet S1**). For example, comparing with other groups, Group A and Group B mostly contained the proteins with a much longer C-terminal domain sharing the same sequence. This fact indicated that some conserved functions might exist in these two groups, which were markedly divaricated from other groups, and that genes in these groups share common founding genes. Accordingly, we found that all the *BZR* genes noted as BAM-like proteins resided in Group A and Group B, while the remaining genes were dispersed in other groups with the annotation BR signaling positive regulator-related proteins (**Supplementary Table S2**).

Of the five major groups, Group A and Group B were sister groups in the phylogenetic tree, with the same for Group C and Group D, implying the distinctive evolutionary closeness among groups. Group D was the biggest clade, containing 23 legume BZR proteins and four *Arabidopsis* BES1/BZR1 homologs (BEH1-4). Group C was the second big clade with 16 members, which included two *Arabidopsis* BZR proteins, BZR1 and BES1. The following clades comprised Group A and Group B, successively, having 11 and seven members, respectively. Compared with BZR proteins in other groups, all of the 18 BZR proteins from Group A and Group B had distinctly longer protein sequences, as well as more introns in relevant transcripts. Group E was the minimal clade, with only one protein—MtBZR1 from *Medicago*.

### Gene Structure and Conserved Motif Analysis of Legume *BZR* Genes in Different Groups

**FIGURE 2:**
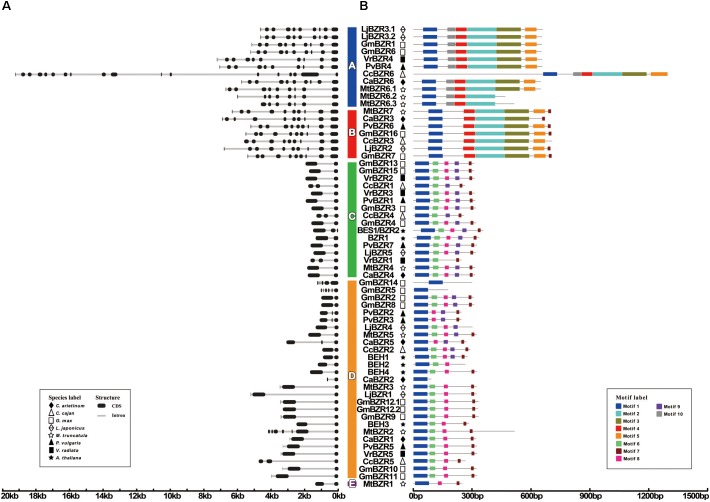
*BZR* gene structures and motifs. Exons are indicated by black boxes; introns are indicated by gray lines. Different motifs are highlighted in different colored boxes with numbers 1 to 10. Phylogenetic groups A–E are indicated. **(A)** Schematic representation of intron-exon composition of *BZR* genes. **(B)** Schematic representation of conserved motifs in *BZR* transcription factors.

We further examined the gene structure of all *BZR* transcripts, including 56 in legumes and six in *Arabidopsis*. There are clear structural patterns which were similar among members within one group but distinct between groups (**Figure 2A**). Compared with *BZR* transcripts in *Arabidopsis* that only had 1∼2 introns, the number of introns in legume *BZR* transcripts varied from 1 to 17. Overall, in legumes, there were 19 transcripts with one intron (33.9%), 16 transcripts with two introns (28.6%), and 21 transcripts with more than three introns (37.5%). Therein, the transcripts in Group A and Group B seemed to possess more introns, six introns at least (as for MtBZR6.3) and 17 introns at most (as for CcBZR6); and, for almost all of them, there were nine introns with only a few exceptions. Conversely, *BZR* transcripts in Group C and Group E had minimal introns, only one or two. The number of introns in Group D exhibited great variation between members, since most transcripts had 1∼2 introns, but a few had more, as in the case of *GmBZR14* (4 introns), *GmBZR5* (5 introns), and *MtBZR2* (6 introns).

Our classification of *BZR* genes in legumes was also verified by the conserved motif analysis of BZR proteins. All of the legume BZR protein sequences were loaded into the MEME analysis tool (Bailey et al., 2009). As a result, a total of 10 statistically significant (e-value less than e-100) conserved motifs were found (**Figure 2B**). The consensus sequences and the amino acid lengths of these conserved motifs are given in **Supplementary Figure S1** and **Table S3**. Motif-1 is a common motif in all BZR proteins, corresponding to the BZR domain, which is the most conserved region of BZR proteins and functions as a DNA binding domain by the presence of an atypical bHLH DNA-binding motif (Yin et al., 2005). However, some motifs are group-specific. Motifs 2 to 5 were specific for Group A and Group B. The NCBI Conserved Domains analysis indicated that they correspond to a BAM-like domain, characteristic of proteins involved in starch breakdown (Monroe et al., 2014). This could explain why *BZR* genes in Group A and Group B have different functional annotations from those in the other groups (**Supplementary Table S2**). In other words, unlike the conventional *BZR* genes, the *BZR* genes in Group A and Group B encode BAM-like proteins, which possess a BZR domain and BAM-like domain concurrently, implying they are likely furnished with extra functions or function-combinations. Motif-6 was specific for Group C and Group D, and had a serine-rich sequence (SxxxSxxxSxxx-SxxxS), considered as the putative phosphorylation site for members of the GSK3 kinase family (Li and Nam, 2002). Motif-9, which is a PEST domain that participates in controlling protein stability (Yin et al., 2005), was found in most of the Group C members and a few Group D members. Being the only member in Group E, MtBZR1 had no group-specific motifs; the sequence motifs were analogous with those of Group C and Group D, but this protein lacked the serine-rich Motif-6 and PEST domain Motif-9.

### Chromosomal Locations and Gene Duplication Events in the *BZR* Gene Family

**FIGURE 3:**
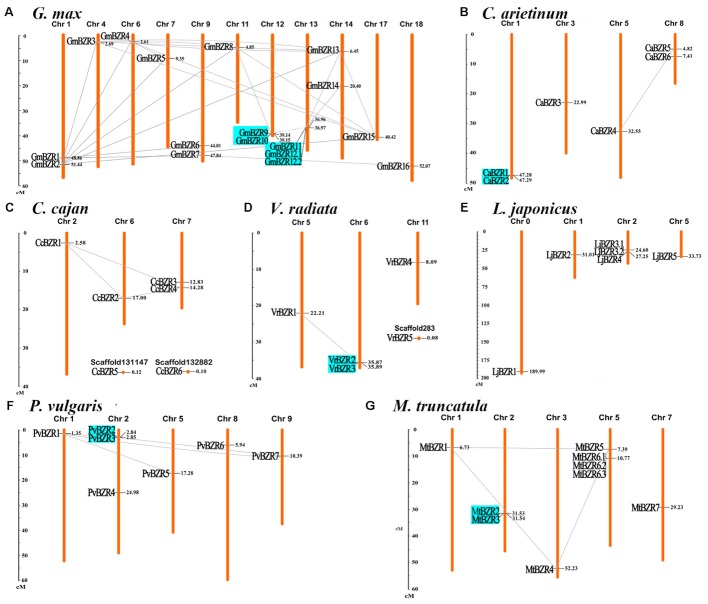
Chromosomal locations and gene duplication events of *BZR* genes. Respective chromosome numbers are indicated at the top of each bar. The scale on the left is in megabases (Mb). *BZR* gene pairs arising from segmental and tandem duplication are linked by lines and marked in cyan, respectively. Distribution of *BZR* genes on the chromosomes of *G. max* **(A)**, *C. arietinum* **(B)**, *C. cajan* **(C)**, *V. radiata* **(D)**, *L. japonicus* **(E)**, *P. vulgaris* **(F)**, *M. truncatula* **(G)**.

The chromosomal distribution of legume *BZR* genes throughout the seven legume genomes was plotted using MapInspect software. Gene duplication events in the *BZR* gene family were also examined, and *BZR* gene-pairs arising from segmental and tandem duplication are linked by lines and marked in cyan, respectively, in **Figure 3**. The pairings created by *BZR* gene duplication are described in detail in **Supplementary Table S4**. *BZR* genes were found to be unevenly distributed on all of the legume chromosomes. The gene expansion of the *BZR* family in legumes mainly resulted from segmental duplication, yet tandem duplication also played a minor role. In total, 37 gene-pairs for segmental duplication and six for tandem duplication were found.

Among these seven legumes, the soybean genome had the most *BZR* genes and the most complicated gene duplication pattern, including segmental and tandem duplication, which is potentially due to its multiple genome duplications or hybridizations (Pagel et al., 2004); however, *BZR* family expansion in the pigeon pea genome resulted only from segmental duplication; while in the lotus genome, there was no evidence of either segmental or tandem duplication of *BZR* genes. Intriguingly, several genes expanded through both tandem and segmental duplication. For example, “*PvBZR2* and *PvBZR3*,” “*GmBZR9* and *GmBZR10*,” and “*GmBZR11* and *GmBZR12*” are gene-pairs representing tandem duplicates; meanwhile, “*PvBZR2* and *PvBZR7*,” “*GmBZR9* and *GmBZR8*,” and “*GmBZR11* and *GmBZR13*” are gene-pairs arising from chromosomal segmental duplication.

*BZR* genes occurring in segmentally duplicated gene pairs can be found in all of the groups (from A to E) on our phylogenetic tree, but these segmental duplication events are more likely to occur within the same phylogenetic group or between sister groups. In about 40% (16 out of 37) of segmentally duplicated gene pairs, both genes were in the same phylogenetic group; the remaining genes paired mostly between sister groups on the phylogenetic tree, such as “A and B” or “C and D.” The only one exception was *MtBZR1* in Group E in *Medicago*, which paired with (i) *MtBZR4* in Group C and (ii) *MtBZR5* in Group D, respectively. Tandemly duplicated genes only emerged in phylogenetic Groups C and D, and merely paired within the same phylogenetic group. Moreover, all of the tandemly duplicated gene-pairs had relatively high sequence similarity (>70%). Therein, “*CaBZR1* and *CaBZR2*,” “*PvBZR2* and *PvBZR3*,” and “*VrBZR2* and *VrBZR3*” had greater than 90% similarity.

**FIGURE 4:**
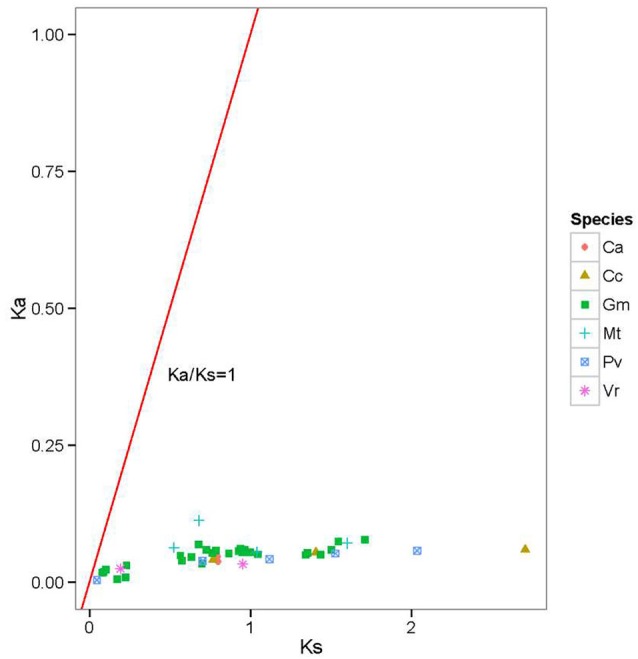
*K*a/*K*s ratios of duplicated legume *BZR* gene pairs. Gene pairs from different species are indicated by different scatter. The y and x axes denote the *Ka* and *Ks* values for each pair and the red line shows a *Ka/Ks* ratio = 1. The detail of the *K*a, *K*s, and *Ka/Ks* listed in **Supplementary Table S4**.

To further understand the evolutionary constraints acting on the legume *BZR* genes, we calculated the *K*a/*K*s ratios for all of the duplicated legume *BZR* gene-pairs. It was shown that all the estimated *K*a/*K*s values were very small (<0.25) (**Figure 4**), suggesting that the *BZR* family has mainly undergone strong purifying selection, reducing divergence after duplication. Using the reciprocal best-hit method, we next identified corresponding gene orthologs present in different legumes (**Supplementary Table S5**). It was noticed that the family members from different species that clustered into one group usually had an orthologous relationship. For example, *MtBZR4*, the only family member from *M. truncatula* in Group C, is orthologous to a cluster of genes in soybean (*GmBZR15*, *4*, *13*, *3*), common bean (*PvBZR1*, *7*), mungbean (*VrBZR2*, *3*), pigeonpea (*CcBZR4*, *1*), chickpea (*CaBZR4*), and birdsfoot trefoil (*LjBZR5*), while their orthologous copies in *Arabidopsis* were *BZR1* and *BZR2*, and all of these BZR members emerged together in phylogenetic Group C. These results further supported the phylogenetic tree, suggesting that the members of the same group usually derived from a common ancestor.

### Synteny Analysis

To explore the evolutionary process of legume *BZR* gene family expansion, we made a comparative analysis of synteny maps among seven legume genomes and the *Arabidopsis* genome. The syntenic blocks were calculated by MCScanX, and the corresponding ideograms were created using Circos (**Supplementary Figure S2A** and **Table S6**). Here, with reference to the *BZR* genes, we discovered 211 syntenic blocks between species, each of which was denoted by a pair of *BZR* genes. We found that, for most of the *BZR* genes, their orthologs were prone to maintain similar and conserved syntenic blocks among different legumes species. For example, six of seven genes (85.71%) in *Medicago* had syntenic blocks with other species; while in mung bean, this proportion was relatively low, only three out of five (60%). In addition, these *BZR* gene-pairs were usually found in either the same phylogenetic group (72%, 152 out of 211) or in phylogenetic sister groups (18%, 39 out of 211).

We further observed that the distribution of *BZR* genes conformed to a high-level pattern of micro- and macro-synteny; that is, if some *BZR* genes in one species were physically close on one chromosome, their corresponding syntenic blocks in another species (if any), would be physically close on a chromosome as well. For instance, *GmBZR1* and *GmBZR2* are a pair of close genes on soybean chromosome 1; they also have parallel syntenic blocks in other species, such as *CaBZR5* and *CaBZR6* blocks in chickpea, and *MtBZR5* and *MtBZR6* blocks in *Medicago*, which are also adjacent in the corresponding genomes (**Supplementary Figure S2B**). In addition, the syntenic blocks of segmentally duplicated *BZR* genes are likely to be conserved as well. For example, the genes *GmBZR7* and *GmBZR16* are segmentally duplicated genes in soybean; their corresponding syntenic blocks in other species are exactly identical, including *CaBZR3* in chickpea, *CcBZR3* in pigeon pea, *LjBZR2* in lotus, *MtBZR7* in *Medicago*, and *PvBZR6* in common bean (**Supplementary Table S6**).

### Expression Profiles of Legume *BZR* Genes Among Different Tissues and Developmental Stages

**FIGURE 5:**
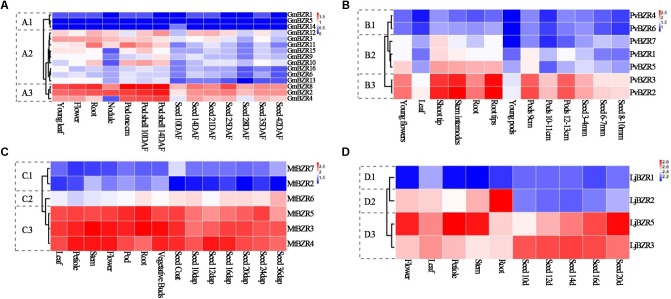
Expression profiles of legume *BZR* genes among different tissues and developmental stages. Clustering of legume *BZR* genes according to their expression profiles among various tissues and different developmental stages in *Glycine max* **(A)**, *Phaseolus vulgaris* **(B)**, *Medicago truncatula* **(C)**, and *Lotus japonicus* **(D)**. 1, 2, and 3 indicate three different clusters of expression patterns, corresponding to low expression (cluster-1), moderate expression with a certain variation (cluster-2), and high expression (cluster-3), respectively. The color scale represents log10 of the average signal values.

Slow rates of protein evolution led us to investigate the expression level of *BZR* genes in different tissues and developmental stages. Expression data from different tissues (nodule, root, stem, leaf, flower, and pod), as well as that from various developmental stages of seeds in soybean, common bean, lotus, and *Medicago*, were collected from public databases. We employed a hierarchical clustering to visualize a global transcription profile of the legume *BZR* genes. As shown in **Figure 5** (see **Supplementary Table S7** for detailed data), *BZR* genes in each legume species could usually be divided into three different clusters, corresponding to low expression (cluster-1), moderate expression with a certain variation (cluster-2), and high expression (cluster-3), respectively. This kind of broad-range variation in expression patterns across various tissues indicated that the members of the legume *BZR* family are expressed either constitutively or in an organ-specific, development-dependent manner and may be involved in organ and tissue differentiation and seed developmental processes.

By comparison with the gene duplication data, we discovered that gene-pairs representing one tandem duplication tended to display similar gene expression clusters, while segmentally duplicated gene-pairs shared fewer similarities (**Supplementary Table S8**). In the genomes of soybean, *Medicago*, common bean, and lotus, there were a total of four pairs of tandemly duplicated genes, in three of which the partner gene was co-located in the same expression cluster, except for one in *Medicago* (*MtBZR2* and *MtBZR3* that were in cluster-1 and cluster-3, respectively). By contrast, we identified 27 gene-pairs arising from segmental duplication in total. Among them, the partners in 16 gene-pairs belonged to different clusters. All these results indicated that tandemly duplicated legume genes tend to maintain similar biological functions to their parental copy due to sharing the same regulatory elements or the slow divergence of their regulation. On the contrary, dispersed segmentally duplicated genes, which are inclined to evolve separate regulatory regions, will more likely have different transcription patterns resulting from recruiting new regulatory elements, which can potentially lead to the divergence of biological functions. Duplicated genes may not necessarily share similar biological functions; they may have neofunctionalization, subfunctionalization, or turned into pseudogenes. In order to determine function of genes, we need further studies to validate how similar or distinct the function of these duplicated genes has become.

When comparing the expression patterns of orthologs among species, on the whole, about half of the orthologous pairs (29 in 65) from different legumes exhibited conserved expression patterns (**Figure 5** and **Supplementary Table S9**). For instance, (i) *MtBZR5* in *Medicago* and its two orthologous copies in common bean (*PvBZR2*, *3*) and two of the three copies in soybean (*GmBZR2*, *8*) were all in cluster-3 that showed high expression in various tissues; (ii) *MtBZR4* in Medicago and its single-copy orthologs *LjBZR5* in lotus and *GmBZR4*, one of four orthologous copies in soybean, displayed highly similar expression patterns and were all in cluster-3.

### Expression Profiles of *Medicago BZR* Genes in Response to Abiotic Stress Treatments

**FIGURE 6:**
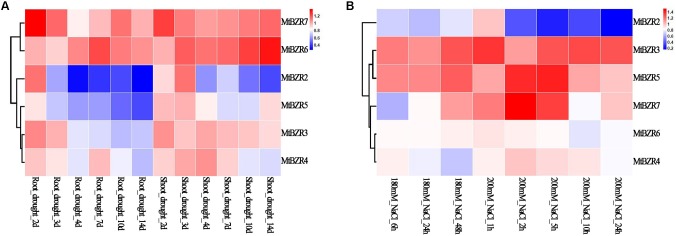
Expression profiles of *Medicago BZR* genes in response to abiotic stress treatments. The fold change in expression of *Medicago BZR* genes identified in **(A)** drought-stressed roots and shoots and **(B)** salt-stressed roots. The color scale represents the fold change in the gene expression value compared with the control.

By using the public transcriptome data from the MtGEA project^14^, we further examined the expression patterns of *Medicago BZR* genes in drought-stressed roots and shoots on the 2nd, 3rd, 4th, 7th, 10th, and 14th day of drought, and in salt-stressed roots upon 180 and 200 mM NaCl treatment. The fold change of gene expression was calculated between abiotic stress treatment and control, and illustrated by a heatmap (**Figure 6** and **Supplementary Table S10**). We observed that *MtBZR2* and *MtBZR5* exhibited significantly lower expression under abiotic stress treatment (fold change < 0.5), while the remaining genes exhibited little or no response.

Among them, *MtBZR5* showed reduced expression when responding to drought treatment, with expression in drought-treated roots (14th day) showing more than twofolddecrease. Meanwhile, *MtBZR2* reacted well to both drought and 200 mM NaCl treatments. With the extension of drought treatment, expression of *MtBZR2* in drought-stressed roots and shoots was down-regulated. On the 14th day of drought treatment, the expression of *MtBZR2* was lowered more than twofold (shoots) and threefold (roots). We also tested the down-regulation of *MtBZR2* in the roots with 200 mM NaCl treatment. In roots subjected to salt stress for 24 h, *MtBZR2* decreased by over fivefold. We then further identified the putative binding sites of upstream regulators of *MtBZR2* by utilizing PlantRegMap. It was found that *MtBZR2* is probably regulated by TFs from other stress-response-related families such as AP2/ERF and MYB (**Supplementary Table S11**), implying a complicated regulatory network underlying abiotic stress tolerance.

### Quantitative PCR Analysis of Soybean *BZR* Genes in Response to Drought Stress Treatments

With the exception of *Medicago*, the transcriptional response of *BZR* genes to abiotic stress is poorly documented in legumes. To further elucidate and validate the response of legume *BZR* genes against abiotic stress, we carried out an expression analysis of soybean *BZR* genes in response to 14-day drought stress treatment, using 2-week-old, soil-grown *G. max* ‘Williams 82’ plants. We selected three soybean *BZR* genes, *GmBZR2*, *GmBZR3*, and *GmBZR8*, for the quantitative PCR experiment. *GmBZR2* and *GmBZR8* are two orthologous copies of the *Medicago* drought stress-responsive gene *MtBZR5*; meanwhile, *GmBZR3* maintains an orthologous relationship with the non-responsive gene *MtBZR4* (**Supplementary Table S5**). In addition, these three orthologous gene pairs also showed chromosomal syntenic relationships and had the most significant protein sequence similarity (**Supplementary Table S6**).

**FIGURE 7:**
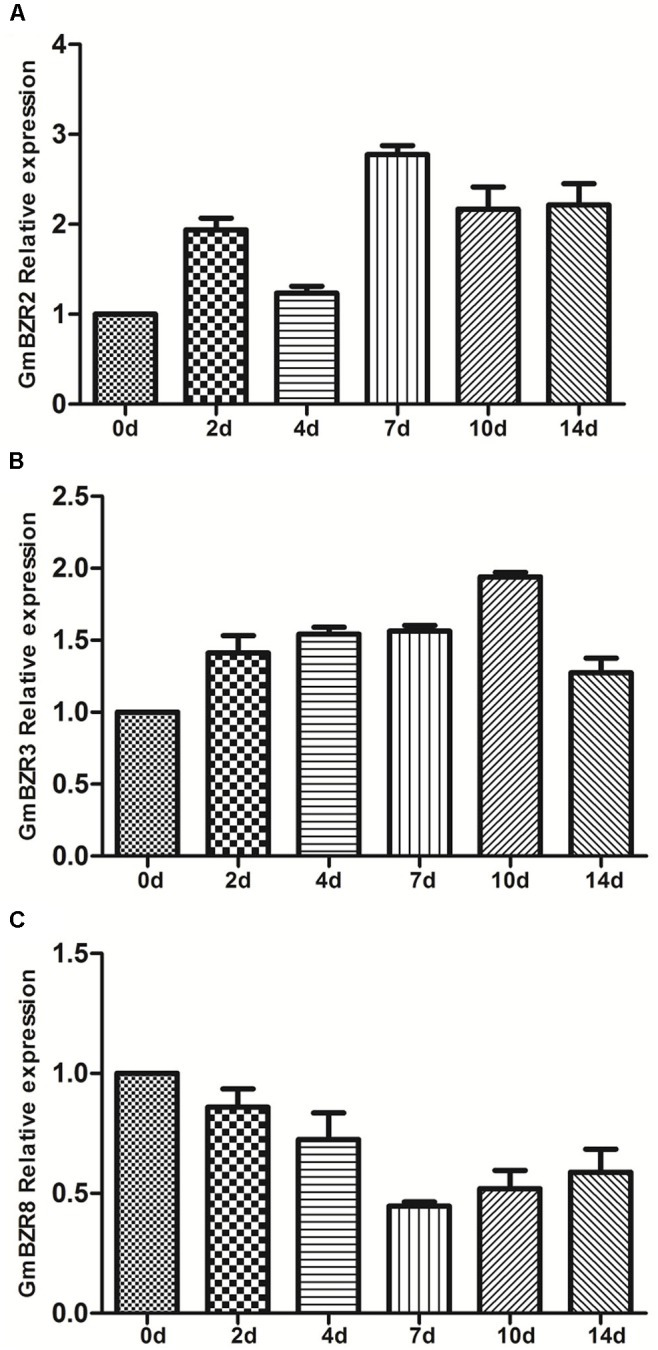
Quantitative PCR analysis of soybean *BZR* genes after drought (0–14 days) treatment. Error bars represent the standard error of the means of three independent replicates. The relative expression level of GmBZR2 **(A)**, GmBZR3 **(B)** and GmBR8 **(C)** was normalized with respect to the GmACTIN gene respectively.

The results showed that there exists some inconsistency between soybean *BZR* genes and their orthologous *Medicago* genes when responding to drought stress treatment (**Figure 7** and **Supplementary Table S12**). For instance, the responses of *GmBZR3* and *GmBZR8* to drought stress treatments were similar to those of their orthologous genes in *Medicago*. With the extension of drought treatment, the gene expression of *GmBZR3*, similarly to that of *MtBZR4*, did not display a response. Expression of *GmBZR8*, similar to that of its ortholog *MtBZR5* in *Medicago*, was down-regulated in drought-stressed roots (7th day, fold change < 0.5). However, the other orthologous copy of *MtBZR5* in soybean, *GmBZR2*, was up-regulated in response to drought stress treatment, such that its expression in drought-treated roots (at the 7th, 10th, and 14th days) showed more than twofold increase. Considering that soybean has a partially diploidized tetraploid genome (Pagel et al., 2004), the drought-stress response of *GmBZR2*, which is distinct from that of its *Medicago* ortholog, is likely related to the mass of segmental duplications and functional divergence in soybean – one of the paralogs may have undergone a change in function, while the other paralog has the same expression as the ortholog. These findings uncovered the complexity of the evolution of gene regulation in legume *BZR* genes, and would be useful for further functional genomics study of *BZR* genes in different legume species.

## Discussion

The *BZR* gene family is an important TF family in regulating plant growth and development and the BZR-mediated abiotic stress response. However, before our study, there had been no genome-wide, in-depth study of the BZR TF family reported in legumes, which are important and widespread crop plants. In this study we identified many members of the *BZR* gene family in legumes, and discovered discrepancies and variations in gene sequence, structure, and conserved motifs. We also identified instances of both the conservation and divergence of the regulation of gene expression, and protein evolution in this family. Expression data analysis and *cis*-regulation prediction further revealed that legume *BZR* genes are potentially intricate participants in regulating the pathways of plant development and resistance. The genome-wide identification and characterization of BZR TF family members in seven legume species is an essential starting point for further exploring the function of this gene family in depth. It is believed that, as the accumulation and extension of data on genomes and transcriptomes continues, there will be a much better understanding of the *BZR* gene family in legumes.

In this work, a total of 52 *BZR* genes were discovered and characterized in seven legume species. BZR family is a small family of important TFs, which are plant-specific proteins without any relationship with gene outside the plant kingdom (Wang et al., 2002). In former publications, there are 6, 15, and 11 BZR genes identified in *A. thaliana*, *Brassica rapa*, and *Zea mays*, respectively (**Table 1**). Except for soybean that has 16 *BZR* genes, the number of *BZR* genes in legumes is comparable to the number of *A. thaliana BZR* genes, which varies from 5 to 7. Compared to other legumes, soybean has a larger genome that assumed the possibility of more *BZR* genes. However, the genome size does not fully explain the fluctuation of the number of *BZR* genes in different plant species. For example, *Brassica rapa* is only about 284 Mb in genome size, but contains 15 *BZR* genes. In contrast, *Zea mays* has a genome of up to 2,135 Mb, but only with 11 *BZR* genes. Therefore, it is very important to identify and characterize new *BZR* genes from diverse plant species so as to elucidate the different expansion mechanisms of *BZR* genes in different plant families.

In our study, phylogenetic analysis divided legume BZR proteins into five major groups with distinctive evolutionary relatedness. Therein, “Groups A and B” and “Groups C and D” are sister groups, respectively; while Group E is unique in the fact that it only has one member (MtBZR1) and is distantly related to the other groups. In terms of gene structure, *MtBZR1* is similar to the members of Group C and Group D; but, as far as protein motifs are concerned, it shows marked discrepancy with the other two groups, with the lack of the serine-rich Motif-6 and PEST domain Motif-9 (**Figure 2B**). By further investigation of *BZR* gene duplication, we found that *MtBZR1* has corresponding segmental-duplication partners in Group C (as *MtBZR4*) and Group D (as *MtBZR5*). These facts indicated that the evolutionary history of MtBZR1 may have included subfunctionalization or non-functionalization with a loss of Motif-6 and Motif-9.

We identified 10 motifs in BZR proteins via conserved motif analysis, in which Motifs 2 to 5, corresponding to a BAM-like domain, characteristic of proteins involved in starch breakdown, were specific for Group A and Group B. Intriguingly, in *Arabidopsis*, this kind of protein with both a BZR DNA binding domain and a BAM-like domain, namely BZR1-BAM proteins, as BAM7 and BAM8 (Reinhold et al., 2011; Soyk et al., 2014), have also been reported. However, reports of BZR1-BAM proteins are rare, except for those in *Arabidopsis*. In addition to the functions of normal BZR proteins, BZR1-BAMs cannot only regulate many genes that react to BRs, but also transmit metabolic signals by binding a ligand in their BAM-like domain, and thus control plant growth and development through cross-talk with BR signaling (Reinhold et al., 2011; Soyk et al., 2014). Therefore, these BZR1-BAM proteins may potentially play some critical functions in the course of legume growth and development.

Genome duplication and subsequent whole-genome fractionation have played important roles in shaping land plant genomes and gene family sizes (Cannon et al., 2004; Jaillon et al., 2007; Schmutz et al., 2010). According to the whole-genome gene duplication analysis of *BZR* genes in *Arabidopsis* and seven legumes, multiple segmental and tandem duplication events also played important roles in elaborating the *BZR* gene family. High-frequency gene duplications may impact the identification of *BZR* gene orthology. For this reason, we adopted an enhanced approach for identification of orthologous gene pairs (Zheng et al., 2005), based on local synteny, protein sequence similarity, and orthologous BLASTN analysis, to predict putative soybean orthologs of drought stress-responsive genes in *Medicago* for further quantitative PCR analysis. Our quantitative PCR results revealed that those genes that react strongly to drought stress treatment in soybean are not completely concordant with the expression behavior of their *Medicago* orthologs, which likely correlates with the complicated history and structure of the soybean genome and the need for further validation. Further studies may validate how similar or distinct the function of these orthologs has become.

## Author Contributions

CL and JC conceived and supervised this study. CL, YL, and LH designed experiments. CL and YL carried out the experiments, and analyzed and interpreted the data. LH, JL, and JC participated in the discussion and provided valuable advice and practical contributions. CL, YL, and JL wrote the manuscript. All authors reviewed, edited, and approved the final manuscript.

## Conflict of Interest Statement

The authors declare that the research was conducted in the absence of any commercial or financial relationships that could be construed as a potential conflict of interest. The reviewer JL and handling Editor declared their shared affiliation.

## Footnotes

1 http://www.icrisat.org/gt-bt/IIPG/Home.html

2 http://nipgr.res.in/CGAP/home.php

3 ftp://ftp.jgi-psf.org/pub/compgen/phytozome/v9.0/Gmax

4 ftp://ftp.kazusa.or.jp/pub/lotus/lotus_r2.5/

5 ftp://ftp.jgi-psf.org/pub/compgen/phytozome/v9.0/Mtruncatula

6 ftp://ftp.jgi-psf.org/pub/compgen/phytozome/v9.0/Pvulgaris

7 http://www.legumeinfo.org/genomes/gbrowse/Vr1.0

8 http://www.softberry.com

9 http://www.atgc-montpellier.fr/phyml/

10 http://mapinspect.software.informer.com/

11 http://plantgrn.noble.org/PvGEA/

12 http://www.soybase.org/

13 http://ljgea.noble.org/v2

14 http://mtgea.noble.org/v3

15 https://cran.r-project.org/web/packages/pheatmap/index.html

## Supplementary Material

DATA SHEET S1 The candidated *BZR* genes in seven legume species identified by both BLASTP and HMM profile searches. (Proteins with partial defect in the N-terminal region are marked in red).

FIGURE S1 The conserved motifs of *BZR* genes.

FIGURE S2 Synteny analysis of legume and Arabidopsis *BZR* genes. **(A)** Chromosomes from different species are depicted as different colored segments. The syntenic counterparts of conserved *BZR* genes between the genomes are interconnected by colored lines. **(B)** The micro- and macro-synteny relationship between GmBZR1/2 and their parallel syntenic blocks in other species.

TABLE S1 Proteins with partial defect in the N-terminal region are marked in red.

TABLE S2 Legume BZR proteins and their related information (MW, molecular weight; Da, dalton; pI, iso-electric point).

TABLE S3 The detailed information of the motifs in BZR proteins.

TABLE S4 The detailed information of the BZR gene duplication pairs.

TABLE S5 The putative orthologs among seven legumes plus *Arabidopsis*.

TABLE S6 The detailed information of the syntenic blocks between seven legume and *Arabidopsis* genomes.

TABLE S7 BZR gene expression data from different tissues and developmental stages in Soybean, Common bean, *Lotus*, and *Medicago*.

TABLE S8 Segmentally/tandemly duplicated BZR gene pairs and the gene expression clusters in which they are located.

TABLE S9 Comparison of BZRs expression from one species to their orthologs expression from the other species (Orthologs with similar expression clusters are marked in red).

TABLE S10 *Medicago* BZR genes expression data (the fold changes between abiotic stress treatments and controls) from drought-stressed roots and shoots, and from salt-stressed roots.

TABLE S11 The putative binding sites of potential upstream regulators of MtBZR2 predicted by PlantRegMap.

TABLE S12 Soybean BZR genes expression data from drought-stressed roots.
