# Supplementary material for: Genome-Wide Identification, Characterization, and Expression Profiling of the Legume BZR Transcription Factor Gene Family
Source: Front Plant Sci. 2018 Sep 19;9:1332. doi: 10.3389/fpls.2018.01332 (PMC6156370; doi:10.3389/fpls.2018.01332)
Supplement: DATA SHEET S1 — The candidated BZR genes in seven legume species identified by both BLASTP and HMM profile searches. (Proteins with partial defect in the N-terminal region are marked in red). [file Data_Sheet_1.PDF]

```
CaBZR1       ............................................................
MtBZR2       ............................................................
PvBZR5       ............................................................
VrBZR5       ............................................................
GmBZR11      ............................................................
GmBZR10      ............................................................
CcBZR5       ............................................................
GmBZR12.1    ............................................................
GmBZR12.2    ............................................................
GmBZR9       ............................................................
LjBZR1       ............................................................
MtBZR3       ............................................................
BEH4         ............................................................
BEH3         ............................................................
CaBZR2       ............................................................
BZR1         ............................................................
BES1/BZR2    ............................................................
CcBZR1       ............................................................
GmBZR15      ............................................................
GmBZR13      ............................................................
PvBZR1       ............................................................
VrBZR3       ............................................................
VrBZR2       ............................................................
MtBZR1       ............................................................
CaBZR4       ............................................................
MtBZR4       ............................................................
GmBZR3       ............................................................
GmBZR4       ............................................................
CcBZR4       ............................................................
PvBZR7       ............................................................
VrBZR1       ............................................................
LjBZR5       ............................................................
BEH1         ............................................................
CaBZR5       ............................................................
MtBZR5       ............................................................
LjBZR4       ............................................................
GmBZR8       ............................................................
GmBZR2       ............................................................
PvBZR2       ............................................................
PvBZR3       ............................................................
CcBZR2       ............................................................
BEH2         ............................................................
GmBZR5       ............................................................
GmBZR14      ............................................................
CaBZR3       ............................................................
MtBZR7       ............................................................
GmBZR7       ............................................................
GmBZR16      ............................................................
PvBZR6       ............................................................
CcBZR3       ............................................................
LjBZR2       ............................................................
LjBZR3.1     ............................................................
LjBZR3.2     ............................................................
CaBZR6       ............................................................
MtBZR6.3     ............................................................
MtBZR6.2     ............................................................
MtBZR6.1     ............................................................
PvBZR4       ............................................................
VrBZR4       ............................................................
GmBZR6       ............................................................
GmBZR1       ............................................................
CcBZR6       MGSSGSKATSSSSSSGSFRKGRSKGHRGFPSYCLGTTSGSRDIDCDDQVCDQSKVNGDDE
```

| | |
|---|---|
| CaBZR1 | ............................................................ |
| MtBZR2 | ............................................................ |
| PvBZR5 | ............................................................ |
| VrBZR5 | ............................................................ |
| GmBZR11 | ............................................................ |
| GmBZR10 | ............................................................ |
| CcBZR5 | ............................................................ |
| GmBZR12.1 | ............................................................ |
| GmBZR12.2 | ............................................................ |
| GmBZR9 | ............................................................ |
| LjBZR1 | ............................................................ |
| MtBZR3 | ............................................................ |
| BEH4 | ............................................................ |
| BEH3 | ............................................................ |
| CaBZR2 | ............................................................ |
| BZR1 | ............................................................ |
| BES1/BZR2 | ............................................................ |
| CcBZR1 | ............................................................ |
| GmBZR15 | ............................................................ |
| GmBZR13 | ............................................................ |
| PvBZR1 | ............................................................ |
| VrBZR3 | ............................................................ |
| VrBZR2 | ............................................................ |
| MtBZR1 | ............................................................ |
| CaBZR4 | ............................................................ |
| MtBZR4 | ............................................................ |
| GmBZR3 | ............................................................ |
| GmBZR4 | ............................................................ |
| CcBZR4 | ............................................................ |
| PvBZR7 | ............................................................ |
| VrBZR1 | ............................................................ |
| LjBZR5 | ............................................................ |
| BEH1 | ............................................................ |
| CaBZR5 | ............................................................ |
| MtBZR5 | ............................................................ |
| LjBZR4 | ............................................................ |
| GmBZR8 | ............................................................ |
| GmBZR2 | ............................................................ |
| PvBZR2 | ............................................................ |
| PvBZR3 | ............................................................ |
| CcBZR2 | ............................................................ |
| BEH2 | ............................................................ |
| GmBZR5 | ............................................................ |
| GmBZR14 | ............................................................ |
| CaBZR3 | ............................................................ |
| MtBZR7 | ............................................................ |
| GmBZR7 | ............................................................ |
| GmBZR16 | ............................................................ |
| PvBZR6 | ............................................................ |
| CcBZR3 | ............................................................ |
| LjBZR2 | ............................................................ |
| LjBZR3.1 | ............................................................ |
| LjBZR3.2 | ............................................................ |
| CaBZR6 | ............................................................ |
| MtBZR6.3 | ............................................................ |
| MtBZR6.2 | ............................................................ |
| MtBZR6.1 | ............................................................ |
| PvBZR4 | ............................................................ |
| VrBZR4 | ............................................................ |
| GmBZR6 | ............................................................ |
| GmBZR1 | ............................................................ |
| CcBZR6 | TYTSGNEIDSDEGKTESFRKVKSDEVPCVPSNIDLEEWGPTASRTGSSSAHSSSNRSLNT |

```
CaBZR1      ............................................................
MtBZR2      ............................................................
PvBZR5      ............................................................
VrBZR5      ............................................................
GmBZR11     ............................................................
GmBZR10     ............................................................
CcBZR5      ............................................................
GmBZR12.1   ............................................................
GmBZR12.2   ............................................................
GmBZR9      ............................................................
LjBZR1      ............................................................
MtBZR3      ............................................................
BEH4        ............................................................
BEH3        ............................................................
CaBZR2      ............................................................
BZR1        ............................................................
BES1/BZR2   ............................................................
CcBZR1      ............................................................
GmBZR15     ............................................................
GmBZR13     ............................................................
PvBZR1      ............................................................
VrBZR3      ............................................................
VrBZR2      ............................................................
MtBZR1      ............................................................
CaBZR4      ............................................................
MtBZR4      ............................................................
GmBZR3      ............................................................
GmBZR4      ............................................................
CcBZR4      ............................................................
PvBZR7      ............................................................
VrBZR1      ............................................................
LjBZR5      ............................................................
BEH1        ............................................................
CaBZR5      ............................................................
MtBZR5      ............................................................
LjBZR4      ............................................................
GmBZR8      ............................................................
GmBZR2      ............................................................
PvBZR2      ............................................................
PvBZR3      ............................................................
CcBZR2      ............................................................
BEH2        ............................................................
GmBZR5      ............................................................
GmBZR14     ............................................................
CaBZR3      ............................................................
MtBZR7      ............................................................
GmBZR7      ............................................................
GmBZR16     ............................................................
PvBZR6      ............................................................
CcBZR3      ............................................................
LjBZR2      ............................................................
LjBZR3.1    ............................................................
LjBZR3.2    ............................................................
CaBZR6      ............................................................
MtBZR6.3    ............................................................
MtBZR6.2    ............................................................
MtBZR6.1    ............................................................
PvBZR4      ............................................................
VrBZR4      ............................................................
GmBZR6      ............................................................
GmBZR1      ............................................................
CcBZR6      SNGFLSRFSLVPGNISFRLSRTTSLGSSRPCPVSSAGLSIFNNEDELNLHQRPASGLINR
```

```
CaBZR1       ............................................................
MtBZR2       ............................................................
PvBZR5       ............................................................
VrBZR5       ............................................................
GmBZR11      ............................................................
GmBZR10      ............................................................
CcBZR5       ............................................................
GmBZR12.1    ............................................................
GmBZR12.2    ............................................................
GmBZR9       ............................................................
LjBZR1       ............................................................
MtBZR3       ............................................................
BEH4         ............................................................
BEH3         ............................................................
CaBZR2       ............................................................
BZR1         ............................................................
BES1/BZR2    ............................................................
CcBZR1       ............................................................
GmBZR15      ............................................................
GmBZR13      ............................................................
PvBZR1       ............................................................
VrBZR3       ............................................................
VrBZR2       ............................................................
MtBZR1       ............................................................
CaBZR4       ............................................................
MtBZR4       ............................................................
GmBZR3       ............................................................
GmBZR4       ............................................................
CcBZR4       ............................................................
PvBZR7       ............................................................
VrBZR1       ............................................................
LjBZR5       ............................................................
BEH1         ............................................................
CaBZR5       ............................................................
MtBZR5       ............................................................
LjBZR4       ............................................................
GmBZR8       ............................................................
GmBZR2       ............................................................
PvBZR2       ............................................................
PvBZR3       ............................................................
CcBZR2       ............................................................
BEH2         ............................................................
GmBZR5       ............................................................
GmBZR14      ............................................................
CaBZR3       ...................................MATDMQRLVGMSEDDEEEMGMGVKD
MtBZR7       ...................................MATDMQRLVGMGEDDEE.MGMGMKD
GmBZR7       ...................................MATDMQRLVGTSEDDEE.MGMDVKD
GmBZR16      ...................................MATDMQRLVGTSEDDEE.MGMDVKD
PvBZR6       ...................................MATDMQRLVGTSEDDEE.MGMDVKD
CcBZR3       ...................................MATDMQRLVGTSEDDEE.MGMDVKD
LjBZR2       ...................................MATDMQRFVGTSEDDEE.MGMGVKD
LjBZR3.1     ..........................................MKNTNDDVS...AQDLDP
LjBZR3.2     ..........................................MKNTNDDVS...AQDLDP
CaBZR6       ..........................................MKSINEDAS...VLNLDP
MtBZR6.3     ..........................................MKNTTEDATS..TQDLDP
MtBZR6.2     ..........................................MKNTTEDATS..TQDLDP
MtBZR6.1     ..........................................MKNTTEDATS..TQDLDP
PvBZR4       ..........................................MKSINDDGS...TQDLDP
VrBZR4       ..........................................MKSINDDGS...TQDLDP
GmBZR6       ..........................................MKSVNDDAS...TQDLDP
GmBZR1       ..........................................MKSINDDAS...TQDVDP
CcBZR6       NETQHRSNLLNSSFVSQVPIQCHEEGSNNLRPNTPALVSPGNLLSSRTRSS...VQDVVR
```

```
CaBZR1       ............................................................
MtBZR2       ............................................................
PvBZR5       ............................................................
VrBZR5       ............................................................
GmBZR11      ............................................................
GmBZR10      ............................................................
CcBZR5       ............................................................
GmBZR12.1    ............................................................
GmBZR12.2    ............................................................
GmBZR9       ............................................................
LjBZR1       ............................................................
MtBZR3       ............................................................
BEH4         ............................................................
BEH3         ............................................................
CaBZR2       ............................................................
BZR1         ............................................................
BES1/BZR2    ............................................................
CcBZR1       ............................................................
GmBZR15      ............................................................
GmBZR13      ............................................................
PvBZR1       ............................................................
VrBZR3       ............................................................
VrBZR2       ............................................................
MtBZR1       ............................................................
CaBZR4       ............................................................
MtBZR4       ............................................................
GmBZR3       ............................................................
GmBZR4       ............................................................
CcBZR4       ............................................................
PvBZR7       ............................................................
VrBZR1       ............................................................
LjBZR5       ............................................................
BEH1         ............................................................
CaBZR5       ............................................................
MtBZR5       ............................................................
LjBZR4       ............................................................
GmBZR8       ............................................................
GmBZR2       ............................................................
PvBZR2       ............................................................
PvBZR3       ............................................................
CcBZR2       ............................................................
BEH2         ............................................................
GmBZR5       ............................................................
GmBZR14      ............................................................
CaBZR3       EDD.........................................................
MtBZR7       DDD.........................................................
GmBZR7       EDD.........................................................
GmBZR16      EDD.........................................................
PvBZR6       EDE.........................................................
CcBZR3       EDD.........................................................
LjBZR2       EDE.........................................................
LjBZR3.1     QSD.........................................................
LjBZR3.2     QSD.........................................................
CaBZR6       QSD.........................................................
MtBZR6.3     QSD.........................................................
MtBZR6.2     QSD.........................................................
MtBZR6.1     QSD.........................................................
PvBZR4       QSD.........................................................
VrBZR4       QSD.........................................................
GmBZR6       QSD.........................................................
GmBZR1       QSD.........................................................
CcBZR6       DGDGTREVPDVNFYSPRIHTDTENFETRHTDRRNGAREPVERNVRFSRTLSVGRLRDRVL
```

```
CaBZR1      ............................................................
MtBZR2      ............................................................
PvBZR5      ............................................................
VrBZR5      ............................................................
GmBZR11     ............................................................
GmBZR10     ............................................................
CcBZR5      ............................................................
GmBZR12.1   ............................................................
GmBZR12.2   ............................................................
GmBZR9      ............................................................
LjBZR1      ............................................................
MtBZR3      ............................................................
BEH4        ............................................................
BEH3        ............................................................
CaBZR2      ............................................................
BZR1        ............................................................
BES1/BZR2   ............................................................
CcBZR1      ............................................................
GmBZR15     ............................................................
GmBZR13     ............................................................
PvBZR1      ............................................................
VrBZR3      ............................................................
VrBZR2      ............................................................
MtBZR1      ............................................................
CaBZR4      ............................................................
MtBZR4      ............................................................
GmBZR3      ............................................................
GmBZR4      ............................................................
CcBZR4      ............................................................
PvBZR7      ............................................................
VrBZR1      ............................................................
LjBZR5      ............................................................
BEH1        ............................................................
CaBZR5      ............................................................
MtBZR5      ............................................................
LjBZR4      ............................................................
GmBZR8      ............................................................
GmBZR2      ............................................................
PvBZR2      ............................................................
PvBZR3      ............................................................
CcBZR2      ............................................................
BEH2        ............................................................
GmBZR5      ............................................................
GmBZR14     ............................................................
CaBZR3      ............................................................
MtBZR7      ............................................................
GmBZR7      ............................................................
GmBZR16     ............................................................
PvBZR6      ............................................................
CcBZR3      ............................................................
LjBZR2      ............................................................
LjBZR3.1    ............................................................
LjBZR3.2    ............................................................
CaBZR6      ............................................................
MtBZR6.3    ............................................................
MtBZR6.2    ............................................................
MtBZR6.1    ............................................................
PvBZR4      ............................................................
VrBZR4      ............................................................
GmBZR6      ............................................................
GmBZR1      ............................................................
CcBZR6      RRSTLSDFTICPLQREREVRDASQDNGRRAGERDTRVSPSGRNAANSSTPRYPQPSTPSS
```

```
CaBZR1      ............................................................
MtBZR2      ............................................................
PvBZR5      ............................................................
VrBZR5      ............................................................
GmBZR11     ............................................................
GmBZR10     ............................................................
CcBZR5      ............................................................
GmBZR12.1   ............................................................
GmBZR12.2   ............................................................
GmBZR9      ............................................................
LjBZR1      ............................................................
MtBZR3      ............................................................
BEH4        ............................................................
BEH3        ............................................................
CaBZR2      ............................................................
BZR1        ............................................................
BES1/BZR2   ............................................................
CcBZR1      ............................................................
GmBZR15     ............................................................
GmBZR13     ............................................................
PvBZR1      ............................................................
VrBZR3      ............................................................
VrBZR2      ............................................................
MtBZR1      ............................................................
CaBZR4      ............................................................
MtBZR4      ............................................................
GmBZR3      ............................................................
GmBZR4      ............................................................
CcBZR4      ............................................................
PvBZR7      ............................................................
VrBZR1      ............................................................
LjBZR5      ............................................................
BEH1        ............................................................
CaBZR5      ............................................................
MtBZR5      ............................................................
LjBZR4      ............................................................
GmBZR8      ............................................................
GmBZR2      ............................................................
PvBZR2      ............................................................
PvBZR3      ............................................................
CcBZR2      ............................................................
BEH2        ............................................................
GmBZR5      ............................................................
GmBZR14     ............................................................
CaBZR3      ........................EDDDYEENGGEHGNV.....................
MtBZR7      ........................EDADYDENGGEHGNV.....................
GmBZR7      ........................EDDDYEENGGEQGNAS....................
GmBZR16     ........................DDDDYEENGGEQGNAS....................
PvBZR6      ........................EDDDYEENGGEQGNA.....................
CcBZR3      ........................EDDEYDENGGEQGNA.....................
LjBZR2      ........................DDDDYDENG.EDGNA.....................
LjBZR3.1    ........................HSSDYLAHSH..........................
LjBZR3.2    ........................HSSDYLAHSH..........................
CaBZR6      ........................HSSDYLAQ............................
MtBZR6.3    ........................HSSDYLPQ............................
MtBZR6.2    ........................HSSDYLPQ............................
MtBZR6.1    ........................HSSDYLPQ............................
PvBZR4      ........................HSSDYLAHPN..........................
VrBZR4      ........................HSSDYLTHPN..........................
GmBZR6      ........................HSSDYLAHP...........................
GmBZR1      ........................HSSDYLAHPN..........................
CcBZR6      LFGIQDYEVETSRSRETRYQDLLEHRSNFLERRRRIRSQVRALQRLGSRFENLSGHDRSC
```

```
CaBZR1      ............................................................
MtBZR2      ............................................................
PvBZR5      ............................................................
VrBZR5      ............................................................
GmBZR11     ............................................................
GmBZR10     ............................................................
CcBZR5      ............................................................
GmBZR12.1   ............................................................
GmBZR12.2   ............................................................
GmBZR9      ............................................................
LjBZR1      ............................................................
MtBZR3      ............................................................
BEH4        ............................................................
BEH3        ............................................................
CaBZR2      ............................................................
BZR1        ............................................................
BES1/BZR2   ............................................................
CcBZR1      ............................................................
GmBZR15     ............................................................
GmBZR13     ............................................................
PvBZR1      ............................................................
VrBZR3      ............................................................
VrBZR2      ............................................................
MtBZR1      ............................................................
CaBZR4      ............................................................
MtBZR4      ............................................................
GmBZR3      ............................................................
GmBZR4      ............................................................
CcBZR4      ............................................................
PvBZR7      ............................................................
VrBZR1      ............................................................
LjBZR5      ............................................................
BEH1        ............................................................
CaBZR5      ............................................................
MtBZR5      ............................................................
LjBZR4      ............................................................
GmBZR8      ............................................................
GmBZR2      ............................................................
PvBZR2      ............................................................
PvBZR3      ............................................................
CcBZR2      ............................................................
BEH2        ............................................................
GmBZR5      ............................................................
GmBZR14     ............................................................
CaBZR3      ............................................................
MtBZR7      ............................................................
GmBZR7      ............................................................
GmBZR16     ............................................................
PvBZR6      ............................................................
CcBZR3      ............................................................
LjBZR2      ............................................................
LjBZR3.1    ............................................................
LjBZR3.2    ............................................................
CaBZR6      ............................................................
MtBZR6.3    ............................................................
MtBZR6.2    ............................................................
MtBZR6.1    ............................................................
PvBZR4      ............................................................
VrBZR4      ............................................................
GmBZR6      ............................................................
GmBZR1      ............................................................
CcBZR6      ILSGQHRNGRCACRIGSRDTNSNDDTNARASISRIVMLAEALFEVLDEIHQQSVVLSSRP
```

| | |
|---|---|
| CaBZR1 | ............................................................ |
| MtBZR2 | ............................................................ |
| PvBZR5 | ............................................................ |
| VrBZR5 | ............................................................ |
| GmBZR11 | ............................................................ |
| GmBZR10 | ............................................................ |
| CcBZR5 | ............................................................ |
| GmBZR12.1 | ............................................................ |
| GmBZR12.2 | ............................................................ |
| GmBZR9 | ............................................................ |
| LjBZR1 | ............................................................ |
| MtBZR3 | ............................................................ |
| BEH4 | ............................................................ |
| BEH3 | ............................................................ |
| CaBZR2 | ............................................................ |
| BZR1 | ............................................................ |
| BES1/BZR2 | ............................................................ |
| CcBZR1 | ............................................................ |
| GmBZR15 | ............................................................ |
| GmBZR13 | ............................................................ |
| PvBZR1 | ............................................................ |
| VrBZR3 | ............................................................ |
| VrBZR2 | ............................................................ |
| MtBZR1 | ............................................................ |
| CaBZR4 | ............................................................ |
| MtBZR4 | ............................................................ |
| GmBZR3 | ............................................................ |
| GmBZR4 | ............................................................ |
| CcBZR4 | ............................................................ |
| PvBZR7 | ............................................................ |
| VrBZR1 | ............................................................ |
| LjBZR5 | ............................................................ |
| BEH1 | ............................................................ |
| CaBZR5 | ............................................................ |
| MtBZR5 | ............................................................ |
| LjBZR4 | ............................................................ |
| GmBZR8 | ............................................................ |
| GmBZR2 | ............................................................ |
| PvBZR2 | ............................................................ |
| PvBZR3 | ............................................................ |
| CcBZR2 | ............................................................ |
| BEH2 | ............................................................ |
| GmBZR5 | ............................................................ |
| GmBZR14 | ............................................................ |
| CaBZR3 | ...............SGMVEIDGGSGIGTAAG............................ |
| MtBZR7 | ...............SGMVEIDGGSGMGTGAG............................ |
| GmBZR7 | ..............VSGMVEIDDGNGIGTATG............................ |
| GmBZR16 | ..............VSGMVEIDGGNGIGTATD............................ |
| PvBZR6 | ...............SGMVGIDGGNEMGTATG............................ |
| CcBZR3 | ...............SGMVEIGGGNGIGTATG............................ |
| LjBZR2 | ...............PGMIEIDDGNGIQTAPG............................ |
| LjBZR3.1 | .........................QLSQPQ............................. |
| LjBZR3.2 | .........................QLSQPQ............................. |
| CaBZR6 | .............................PQ............................. |
| MtBZR6.3 | .............................PQ............................. |
| MtBZR6.2 | .............................PQ............................. |
| MtBZR6.1 | .............................PQ............................. |
| PvBZR4 | .......................SNPHPHPH............................. |
| VrBZR4 | .......................SNPQPHPH............................. |
| GmBZR6 | ..........................QPHPQ............................. |
| GmBZR1 | .......................SHPEPHPQ............................. |
| CcBZR6 | SVSSIGSVPAPNEVVESLPVKLYTKLHKHQEDPVQCYICLVEYEDGDSMRVLPCHHEFHT |

```
CaBZR1       ............................................................
MtBZR2       ............................................................
PvBZR5       ............................................................
VrBZR5       ............................................................
GmBZR11      ............................................................
GmBZR10      ............................................................
CcBZR5       ............................................................
GmBZR12.1    ............................................................
GmBZR12.2    ............................................................
GmBZR9       ............................................................
LjBZR1       ............................................................
MtBZR3       ............................................................
BEH4         ............................................................
BEH3         ............................................................
CaBZR2       ............................................................
BZR1         ............................................................
BES1/BZR2    ............................................................
CcBZR1       ............................................................
GmBZR15      ............................................................
GmBZR13      ............................................................
PvBZR1       ............................................................
VrBZR3       ............................................................
VrBZR2       ............................................................
MtBZR1       ............................................................
CaBZR4       ............................................................
MtBZR4       ............................................................
GmBZR3       ............................................................
GmBZR4       ............................................................
CcBZR4       ............................................................
PvBZR7       ............................................................
VrBZR1       ............................................................
LjBZR5       ............................................................
BEH1         ............................................................
CaBZR5       ............................................................
MtBZR5       ............................................................
LjBZR4       ............................................................
GmBZR8       ............................................................
GmBZR2       ............................................................
PvBZR2       ............................................................
PvBZR3       ............................................................
CcBZR2       ............................................................
BEH2         ............................................................
GmBZR5       ............................................................
GmBZR14      .......................................MQSNVHCLPASCPFPFPFVSA
CaBZR3       ............................................................
MtBZR7       ............................................................
GmBZR7       ............................................................
GmBZR16      ............................................................
PvBZR6       ............................................................
CcBZR3       ............................................................
LjBZR2       ............................................................
LjBZR3.1     ............................................................
LjBZR3.2     ............................................................
CaBZR6       ............................................................
MtBZR6.3     ............................................................
MtBZR6.2     ............................................................
MtBZR6.1     ............................................................
PvBZR4       ............................................................
VrBZR4       ............................................................
GmBZR6       ............................................................
GmBZR1       ............................................................
CcBZR6       TCIDKWLKEIHRPVNQLASNITVLHACDDTLFTFQRKKGKETFQLQCKLRETSVVADPMQ
```

```
                                                                 1       .
CaBZR1        .................................................M.... TLGT.
MtBZR2        .................................................M.... TSGT.
PvBZR5        .................................................M.... TSGT.
VrBZR5        .................................................M.... TSGT.
GmBZR11       .................................................M.... TSGT.
GmBZR10       .................................................M.... TSGT.
CcBZR5        .................................................M.... TSGT.
GmBZR12.1     .................................................M.... TSVA.
GmBZR12.2     .................................................M.... TSVA.
GmBZR9        .................................................M.... TSGA.
LjBZR1        .................................................M.... TSGT.
MtBZR3        .................................................M.... TSGT.
BEH4          .................................................M.... TSGT.
BEH3          .................................................M.... TSGT.
CaBZR2        .................................................M.... TSGT.
BZR1          .................................................M.... TSDGA
BES1/BZR2     ................................MKRFFYNSSEEERKKKAMSSKK TSDGA
CcBZR1        .................................................M.... ASDGA
GmBZR15       .................................................M.... ADDGA
GmBZR13       .................................................M.... VDDGA
PvBZR1        .................................................M.... ASDGA
VrBZR3        .................................................M.... ASDGA
VrBZR2        .................................................M.... TSDGA
MtBZR1        .................................................M.... ASDRE
CaBZR4        .................................................M.... ASDGA
MtBZR4        .................................................M.... ASDGA
GmBZR3        .................................................M.... TSDGA
GmBZR4        .................................................M.... TSDGA
CcBZR4        .................................................M.... TSDGA
PvBZR7        .................................................M.... TSDGA
VrBZR1        .................................................M.... TSDGA
LjBZR5        .................................................M.... PSDGA
BEH1          .................................................M.... TASGG
CaBZR5        .................................................M.... TGGG.
MtBZR5        .................................................M.... TGGG.
LjBZR4        .................................................M.... AGGGG
GmBZR8        .................................................M.... TGGG.
GmBZR2        .................................................M.... TGGG.
PvBZR2        .................................................M.... TGGG.
PvBZR3        .................................................M.... TGGG.
CcBZR2        .................................................M.... TGGG.
BEH2          .................................................M.... AAGGG
GmBZR5        .................................................M.... TGGGS
GmBZR14       SKLPPPLFSLLHLILAQERKKLQSSHKRKQISFTSFAFDGRRGVETNKLMFAGT TGGGS
CaBZR3        ................................................DNSFQQHHQFQE
MtBZR7        ................................................DNRFQQHHEFQE
GmBZR7        ................................................DNRFQQHQQFQE
GmBZR16       ................................................DNRFQQHQQFQE
PvBZR6        ................................................DNRFPQHQQFHE
CcBZR3        ................................................DHRFGQNQQFQE
LjBZR2        ................................................DNRFQQHQQFQE
LjBZR3.1      ................................................PRRPRGFAAAAA
LjBZR3.2      ................................................PRRPRGFAAAAA
CaBZR6        ................................................PRRPRGFAAAAM
MtBZR6.3      ................................................PRRLRGFAATAA
MtBZR6.2      ................................................PRRLRGFAATAA
MtBZR6.1      ................................................PRRLRGFAATAA
PvBZR4        ................................................TRRPRGFAAAAA
VrBZR4        ................................................PRRPRGFAAAAA
GmBZR6        ................................................PRRPRGFAAAAA
GmBZR1        ................................................PRRPRGFAAAPV
CcBZR6        DLVDLPCWIPLGLKQKVEGKKREQKKRNQKMRVNAFHEKPSSVLVLNLPRRPRGFAAATA
```

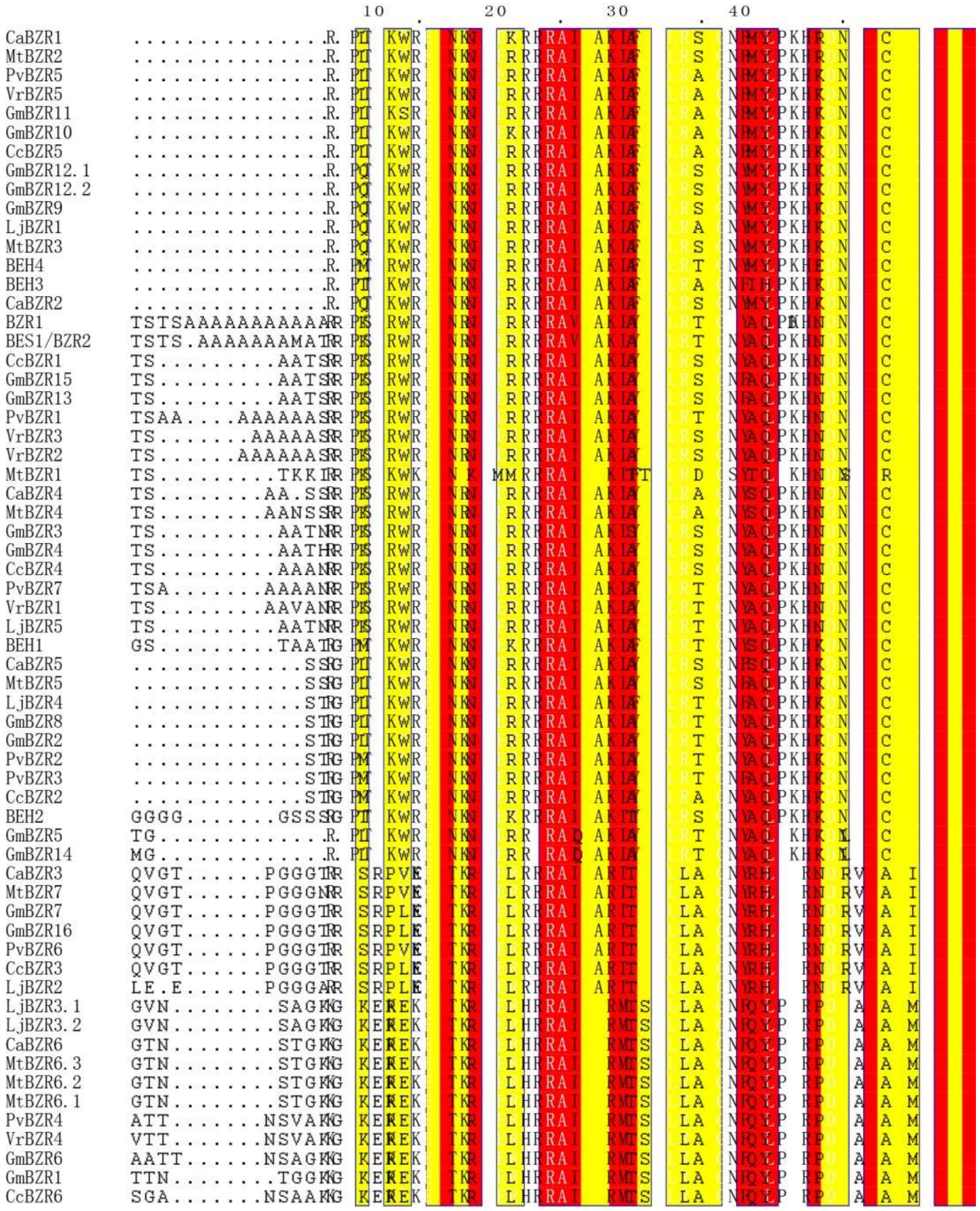
10
20
30
40
CaBZR1
MtBZR2
PvBZR5
VrBZR5
GmBZR11
GmBZR10
CcBZR5
GmBZR12.1
GmBZR12.2
GmBZR9
LjBZR1
MtBZR3
BEH4
BEH3
CaBZR2
BZR1
BES1/BZR2
CcBZR1
GmBZR15
GmBZR13
PvBZR1
VrBZR3
VrBZR2
MtBZR1
CaBZR4
MtBZR4
GmBZR3
GmBZR4
CcBZR4
PvBZR7
VrBZR1
LjBZR5
BEH1
CaBZR5
MtBZR5
LjBZR4
GmBZR8
GmBZR2
PvBZR2
PvBZR3
CcBZR2
BEH2
GmBZR5
GmBZR14
CaBZR3
MtBZR7
GmBZR7
GmBZR16
PvBZR6
CcBZR3
LjBZR2
LjBZR3.1
LjBZR3.2
CaBZR6
MtBZR6.3
MtBZR6.2
MtBZR6.1
PvBZR4
VrBZR4
GmBZR6
GmBZR1
CcBZR6

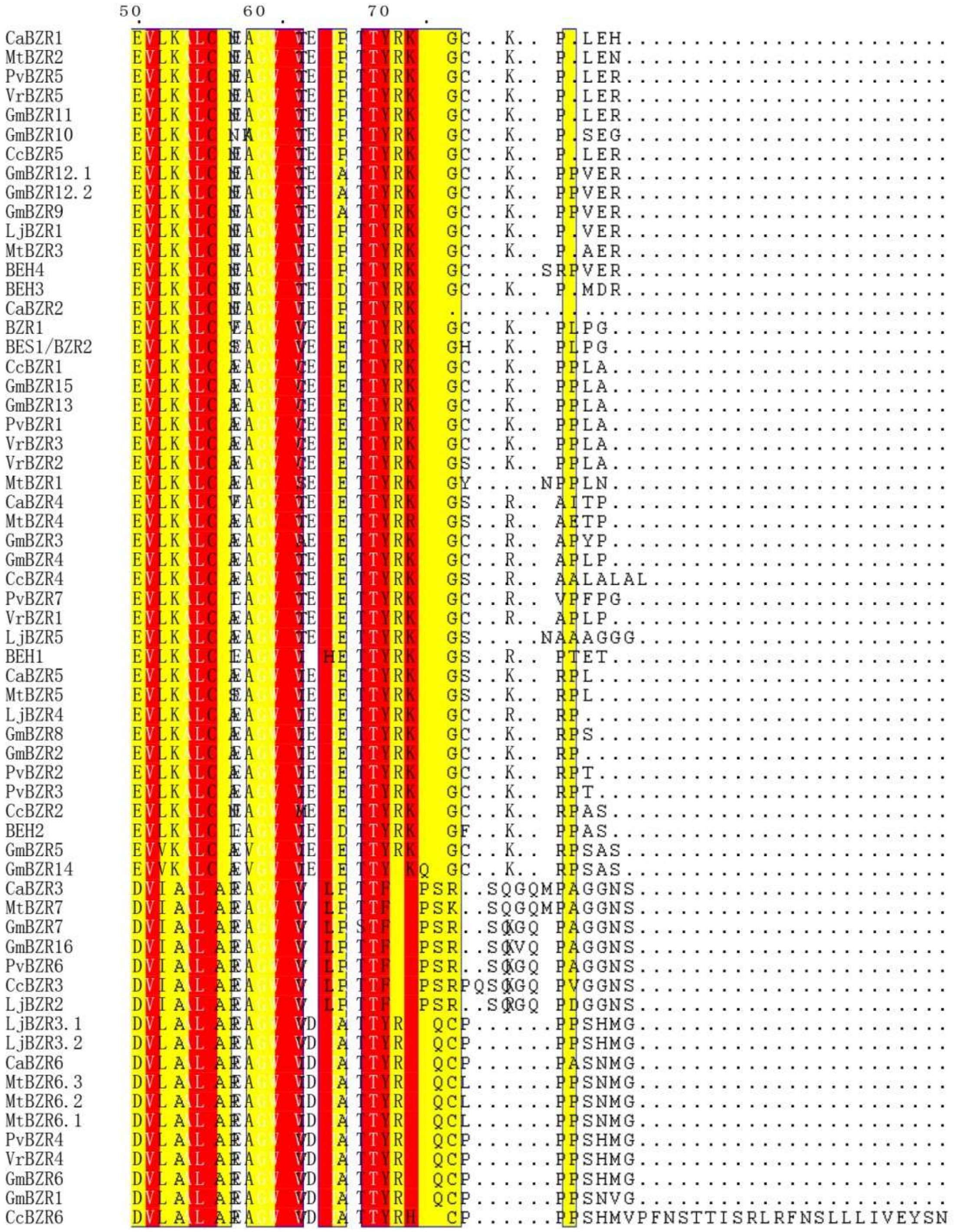
50
60
70
CaBZR1
MtBZR2
PvBZR5
VrBZR5
GmBZR11
GmBZR10
CcBZR5
GmBZR12.1
GmBZR12.2
GmBZR9
LjBZR1
MtBZR3
BEH4
BEH3
CaBZR2
BZR1
BES1/BZR2
CcBZR1
GmBZR15
GmBZR13
PvBZR1
VrBZR3
VrBZR2
MtBZR1
CaBZR4
MtBZR4
GmBZR3
GmBZR4
CcBZR4
PvBZR7
VrBZR1
LjBZR5
BEH1
CaBZR5
MtBZR5
LjBZR4
GmBZR8
GmBZR2
PvBZR2
PvBZR3
CcBZR2
BEH2
GmBZR5
GmBZR14
CaBZR3
MtBZR7
GmBZR7
GmBZR16
PvBZR6
CcBZR3
LjBZR2
LjBZR3.1
LjBZR3.2
CaBZR6
MtBZR6.3
MtBZR6.2
MtBZR6.1
PvBZR4
VrBZR4
GmBZR6
GmBZR1
CcBZR6

```
                                     80       90        100        110        120
CaBZR1       ................MDLVSG .SAASPCSSYHQSPCASYNPSPVSSSFPSPSSSPYTT
MtBZR2       ................MDMVSG .SAASPCSSYH........PSPGSSSFPSPSSSPYAA
PvBZR5       ................MDLVSG .SAASPCSSYHPSPCASYNPSPGSSSFPSPSSSPYTN
VrBZR5       ................MDLVSG .SAASPCSSYHPSPCASYNPSPGSSSFPSPSSSPYTN
GmBZR11      ................MDLVSG .SAASPCSSYHPSPCASYNPSPGSSSFPSPSSSPYTQ
GmBZR10      ................MELVSG SAAASPCSSYHPSPCASYNPSPGSS......SPYYTQ
CcBZR5       ................MDLVSG .SAASPCSSYHPSPCASYNPSPGSSSFPSPSSSPYTT
GmBZR12.1    ................MDLVSG .AAASPCSSYHPSPCASYNPSPGSSCLPSPRASPFPP
GmBZR12.2    ................MDLVSG .AAASPCSSYHPSPCASYNPSPGSSCLPSPRASPFPP
GmBZR9       ................MDLVSG .AAASPCSSYHPSPCASYNPSPGSSCLPSPRASPYPP
LjBZR1       ................MDVVSC .AAPSPCSSYHPSPCASYNPSPGSSSFPSPRSSPYAV
MtBZR3       ................IDVISG .TMGSPCSSYHVSPCASYNPSPGSSSFPSPRSS.HAV
BEH4         ................MELGSG .ATASPCSSYQPSPCASYNPSPGSSNFMSPASSSFAN
BEH3         ................MDLMNG .TSASPCSSYQHSPRASYNPSPSSSSFPSP......T
CaBZR2       ......................................................
BZR1         .................ELAST SRVTPYS.SQNQSPLSSAFQSPIPSYQVSPSSSSFPS
BES1/BZR2    .................DMASS SRATPYS.SHNQSPLSSTFDSPILSYQVSPSSSSFPS
CcBZR1       .................NGASS MRSIPFSSSQNPSPLSSSFPSPIPSYQVSPSSSTFPS
GmBZR15      .................NGASS MRNIPFSSSQNPSPLSSSFPSPIPSYQVSPSSSSLPS
GmBZR13      .................NGASS MRNITFSSSQNPSPLSSSFPSPIPSYQVSPSSSSFPS
PvBZR1       .................NGASS MRNIPFCSSQNQSPFSSSYPSPIPSYQVSPSSSSFPS
VrBZR3       .................NGASN MRNVPFCSSQSPSPFSSSYPSPIPSYQVSPSSSSFPS
VrBZR2       .................NGASS MRNILFSSSQNPSPFSSSHPSPIPSYQASPSSSSFPS
MtBZR1       .................NLAST .....FSHSQNPCLICPSFPTSIPSYQFTPQP.....
CaBZR4       ................GDGS TCTRNNPFS.SQNPSPLSSSFPSPIHSYQVSPSSSSFPS
MtBZR4       ................GDGA NFNRNNPFS.SQNLSPLSSSFPSPIPSYQVSPSSSSFPS
GmBZR3       ................GDGVST TRNTPFS.SQNPSPLSSSFPSPIPSYQVSPSSSSFPS
GmBZR4       ................GDGVST TRNTPFS.SQNPSPLSSSFPSPIPSYQVSPSSSSFPS
CcBZR4       ...............AGDGAST SRNTPFS.SQNPS........PIPSYQVSPSSSSFPS
PvBZR7       .................DGADT IRNTPFS.SQNLSPLSSSFPSPIPSYQVSPSSSSFPS
VrBZR1       .................DGVST TRNTPFS.SQNPSPLSSSFPSPIPSYQVSPSSSSFPS
LjBZR5       ...............AGDGAST TRNTPFS.SQNPSPLSSSFPSPIPSYQVSPTSSSFPS
BEH1         ............................TVPCS.SIQLSPQSSAFQSPIPSYQASPSSSSYPS
CaBZR5       ................PNEITG PPNMSACSSMQPSPQS....................
MtBZR5       ................PNEMTG PTNMSACSSMQPSPQSSSFPSPQSSSFPSPIPSYPTS
LjBZR4       ................PIEITG PPNMSACSSIQPSPQSSSFPSPVPSYHASPTSSSFPS
GmBZR8       ................ASEITG VANISACSSIQPSPQSSSYPSPVPSYHASPTSSSFPS
GmBZR2       ................TSEITG PLNLSACSSIQASPQSSSYPSPVPSYHASPTSSSFPS
PvBZR2       ................GSEITG PPNISA..............................
PvBZR3       ................GSEITG PPNISA..............................
CcBZR2       ................GSEI G.....AMDSSIQASPQSSSFPSPVPSYQASPTSSSFPS
BEH2         ..................DISG PTNFSTNSSIQPSPQSSAFPSPAPSYHGSPVSSSFPS
GmBZR5       ..................EIEG TTNIR...............................
GmBZR14      ..................EIEG TTNISCLLSFVSTFLCVPVMLEYKIMSFYISCLLVRQ
CaBZR3       ....TVVTSSSSHVPSQQPPSV LKGVASGYRSPLEYSACPSKGVFIPSPSPYDLSSSSR
MtBZR7       ....AVVTSSSSHVPSQQPPSV LKGVASGYGSPLEYNACPMKDVFIPSPSPYDLSSTSR
GmBZR7       ....TIVTSSSSLAASQQTPSA LRGVASGYRSPLEYNACQTKGVFMPTPSPYDLSSSSR
GmBZR16      ....TIVTSSSSHAASQQTPSA LRGVASGYRSPLEYNACQTKSVFMPTPSPYGLSSSSR
PvBZR6       ....TIVTSSSSHVASQQTPSP LRGVASGHRSPLEYSAS..KGVFMPTPSPYNLSSTSR
CcBZR3       ....TIVTSSS.HVASQQTPSA LRGVASGYRSPLEYNACQTKGVLMPTPSPYDLSSSSR
LjBZR2       ....APVTSSS.QVPSQQTPSA LRGVASGYGSPLEYNACQMKGVFMPAPSPYDLSSSSR
LjBZR3.1     .......SFAAARSVETQLSAG LR..ACSVKETLENQPP.....VLRIDECLSPASIDS
LjBZR3.2     .......SFAAARSVETQLSAG LR..ACSVKETLENQPP.....VLRIDECLSPASIDS
CaBZR6       .......SFAAR.SVESQLSSG LR..TCSVQETLENQPPV....VLRIDECLSPASIDS
MtBZR6.3     .......SFAAR.SVESQBSTG LR..TCSVKETLENQSP.....GLRIDECVSPASIDS
MtBZR6.2     .......SFAAR.SVESQBSTG LR..TCSVKETLENQSP.....GLRIDECVSPASIDS
MtBZR6.1     .......SFAAR.SVESQBSTG LR..TCSVKETLENQSP.....GLRIDECVSPASIDS
PvBZR4       .......SFAAK.SVESQLSSG LR..NCSVKETIENQTS.....VLRIDECLSPASIDS
VrBZR4       .......SFAAK.SVESQLSSG LR..NCSVKETIENQTS.....VLRIDECLSPASIDS
GmBZR6       .......SFAAR.SVESQLSSG LR..NCSVKETIENQTS.....VLRIDECLSPASIDS
GmBZR1       .......SFAAR.SVESQLSSG LR..NCSVKETIENQTA.....VLRIDECLSPASIDS
CcBZR6       LLMVYGGSLAAR.SVESQLSSG LR..NCSVKETIENQTS.....VLRIDECLSPASIDS
```

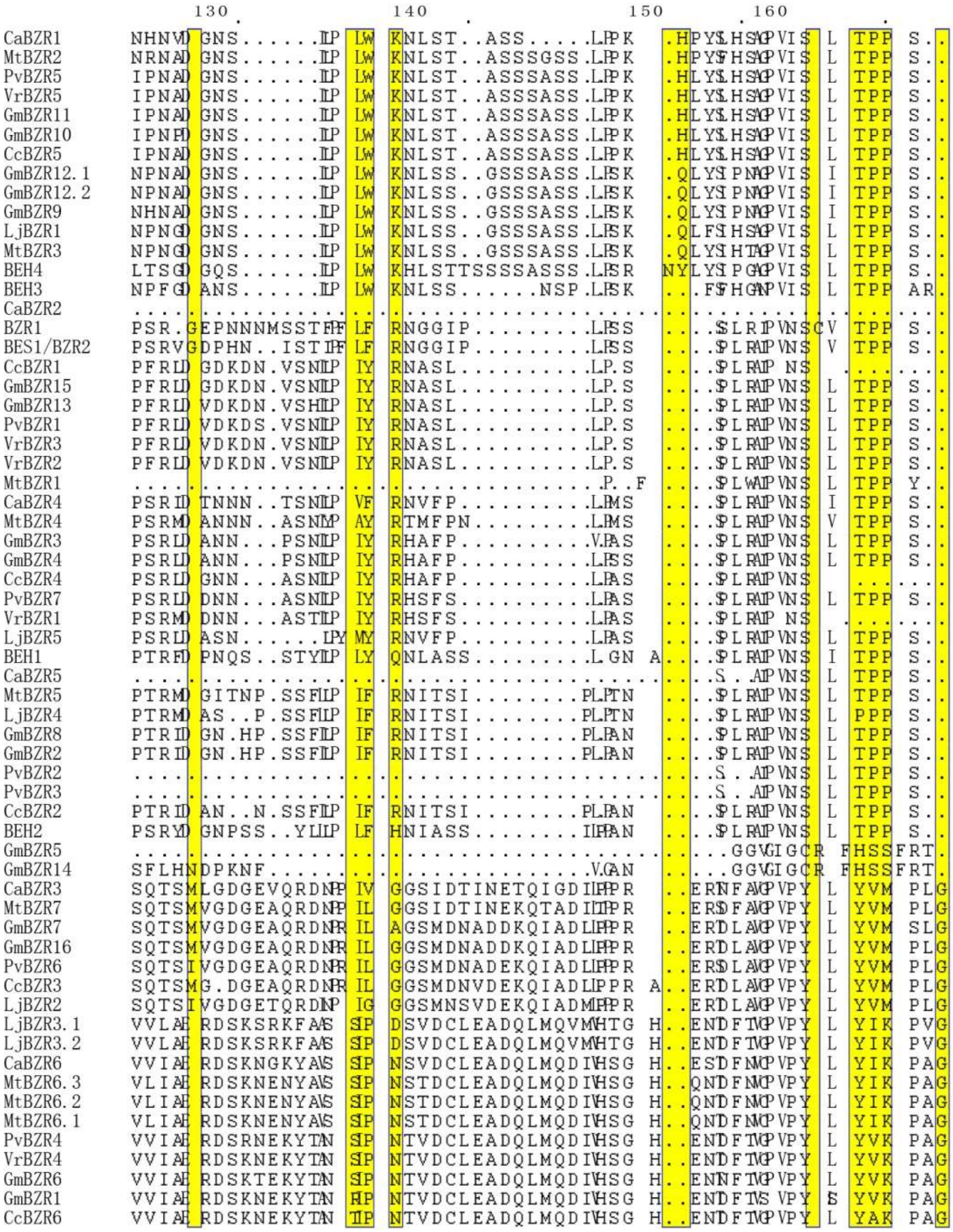
130
140
150
160
CaBZR1
MtBZR2
PvBZR5
VrBZR5
GmBZR11
GmBZR10
CcBZR5
GmBZR12.1
GmBZR12.2
GmBZR9
LjBZR1
MtBZR3
BEH4
BEH3
CaBZR2
BZR1
BES1/BZR2
CcBZR1
GmBZR15
GmBZR13
PvBZR1
VrBZR3
VrBZR2
MtBZR1
CaBZR4
MtBZR4
GmBZR3
GmBZR4
CcBZR4
PvBZR7
VrBZR1
LjBZR5
BEH1
CaBZR5
MtBZR5
LjBZR4
GmBZR8
GmBZR2
PvBZR2
PvBZR3
CcBZR2
BEH2
GmBZR5
GmBZR14
CaBZR3
MtBZR7
GmBZR7
GmBZR16
PvBZR6
CcBZR3
LjBZR2
LjBZR3.1
LjBZR3.2
CaBZR6
MtBZR6.3
MtBZR6.2
MtBZR6.1
PvBZR4
VrBZR4
GmBZR6
GmBZR1
CcBZR6

```
              170         180                   190       200
CaBZR1       .........S TARTPRMNSDWDDRSARPG..WTG......QHYSFLPSSGPPSPGRQIV
MtBZR2       .........S TSRTPRLNADFDDQSARPG..WTG......QHYSFLPSSGPPSPARQIV
PvBZR5       .........S TARTPRINAEWEDQSVRPG..WTRQ.....QHYSFLPSSSPPSPGRQVV
VrBZR5       .........S TARTPRINAEWEDQSVRPG..WTRQ.....QHYSFLPSSSPPSPGRQVV
GmBZR11      .........S TSRTPRINVEWDEQSARPG..WTRQ.....QHYSFLPSSSPPSPGRQVV
GmBZR10      .........S TARTPRINAEWDEQSARPGPGWTRQ.....QHYSFLPSSSPPSPGRQVV
CcBZR5       .........S TARTPRTNVD.......................................
GmBZR12.1    .........S SSRKPQIRADWEDQSNCPT..AWGG.....PAYTFVPSSTPPSPGRQVA
GmBZR12.2    .........S SSRKPQIRADWEDQSNCPT..AWGG.....PAYTFVPSSTPPSPGRQVA
GmBZR9       .........S SSRKPRINADWEDLSTRPA..AWGG.....PAYTFLPSSTPPSPGRQVA
LjBZR1       .........S TARTPRMQADWEDQSTRP...GWGG.....QQYPFLPSSTPPSPGRQVV
MtBZR3       .........S TARSPQRKADWEDQSTRP...GWGG.....QQYSFQPSSTPPSPGRQVL
BEH4         .........S TARTPRMNTDWQQLNN.................SFFVSSTPPSPTRQII
BEH3         .........S TRDQVTIP.........................................
CaBZR2       .......... .................................................
BZR1         .........S TSKNPK.PLPNWESIAKQSMAIA.KQSMASFNYPFYAVSAPASPTHRH.
BES1/BZR2    .........S TSRNPK.PLPTWESFTKQSMSMAAKQSMTSLNYPFYAVSAPASPTHHR.
CcBZR1       .......... .................................................
GmBZR15      .........S TSRNSK.PIPTWESIAKESMASFN........YPFFAASAPASPTHR..
GmBZR13      .........S TSRNPK.PIPTWESIAKESMASFS........YPFFAASAPASPTHR..
PvBZR1       .........S TSRNPK.PIPTWESIAKESMASFN........YPLFAASAPASPTHR..
VrBZR3       .........S TSRNPK.PIPTWESIAKESMTSFN........YPLFAASAPASPTHR..
VrBZR2       .........S TSRNPK.PIPTWESIAKESMTSFN........YPLFAASAPASPTHR..
MtBZR1       .........S TSTNLT.PIPAWDSISKESMAFFS........YPFVASSTPASPTHQ..
CaBZR4       .........S TSRNPKPMIPTWDSIAKASVASFN........HPFFAASAPTSPSHR..
MtBZR4       .........S TSRNPKPMIPTWESIAKASGTSFN........HPFFAASAPTSPSHR..
GmBZR3       .........S TSRNPK.PIPTWDSIAKASMASSFNHSH....HPFFAASAPASPTHR..
GmBZR4       .........S TSRNPK.PIPTWDSIAKASMASSFNHSH....HPFFAASAPASPTHR..
CcBZR4       .......... ...........................................PTHR..
PvBZR7       .........S TSRNPK.PIPTWDSIVKASMASSFNHHHQ..NHPLFAASAPASPTHR..
VrBZR1       .......... .................................................
LjBZR5       .........S TSRNNPKPIPTWDSIAKASMAAASFN......HPLFAASAPASPSHR..
BEH1         .........S RRSNPR..LPRWQSSN...................FPVSAPSSPTRR..
CaBZR5       .........S RSSKRK...ADFESLSNGSLNSSFR.......HPLFATSAPSSPSRR..
MtBZR5       .........S RSSKRK...ADFESLCNGSFNSSFR.......HPLFATSAPSSPSRR..
LjBZR4       .........S RSSKRK...ADFEPFSNGSFN.AFR.......HPLFATSAPSSPSRR..
GmBZR8       .........S RSSKRK...ADFDSLHN....ASLR.......HPLFATSAPSSPSRR..
GmBZR2       .........S RSSKRK...ADFD.........SLR.......HPLFATSAPSSPTRR..
PvBZR2       .........S RTSKRK...ANFDSLSH....ASFR.......HPLFATSAPSSPSRR..
PvBZR3       .........S RTSKRK...ANFDSLSH....ASFR.......HPLFATSAPSSPSRR..
CcBZR2       .........S RASKRK...ANFH....................PLFATSAPSSPTRR..
BEH2         .........S TSRGSKRKLTSEQLPNGGSLHVLR........HPLFAISAPSRLVVL..
GmBZR5       ........TQ SG...............................................
GmBZR14      ........TQ SG...............................................
CaBZR3       VINIKCELVD DALLKQLRVLKSANVDGVMVDCWWGIVEAHAPQEYNWNGYKRLFQMLRE
MtBZR7       VINIKCELVD DGILKQLRVLKSANVDGVMVDCWWGIVEAHAPQEYNWNGYKRLFQMVRE
GmBZR7       VINIKCELVD DGLLKQLRVLKSVHVDGVMVDCWWGIVEAHAPQEYNWNGYKRLFQMVRE
GmBZR16      VINIKCELVD DGLLKQLKVLKSVHVDGVMVDCWWGIVEAHAPQEYNWNGYKRLFQMVRE
PvBZR6       VINIKCELVD DGLLKQLRVLKSVHVDGVMVDCWWGIVEAHAPQEYTWNGYKRLFQMVRE
CcBZR3       VINIKCELVD DGLLKQLRVLKSVHVDGVMVDCWWGIVEAHAPQEYNWNGYKRLFQMVRE
LjBZR2       VINIKCELVD DGLLKQLRVLKSINVDGVMVDCWWGIVEAHAPQEYNWNGYKRLFQMVRE
LjBZR3.1     IINKFCQLID EGIRQELIHIKSLNIDGVVVDCWWGIVEGWSPQKYVWSGYRELFNIIRE
LjBZR3.2     IINKFCQLID EGIRQELIHIKSLNIDGVVVDCWWGIVEGWSPQKYVWSGYRELFNIIRE
CaBZR6       IINKFCQLID EGIRQELIHIKSLNIDGVVVDCWWGIVEGWSPQKYVWSGYRELFNIIRE
MtBZR6.3     IINKFCQLMD EGIRQELIHIKSLNIDGVVVDCWWGIVEGWNSQKYEWSGYRELFSIIRE
MtBZR6.2     IINKFCQLMD EGIRQELIHIKSLNIDGVVVDCWWGIVEGWNSQKYEWSGYRELFSIIRE
MtBZR6.1     IINKFCQLMD EGIRQELIHIKSLNIDGVVVDCWWGIVEGWNSQKYEWSGYRELFSIIRE
PvBZR4       IINKFCQLID EGIREELMHMKSLNVDGVVVDCWWGIVEGWSSQKYVWSGYRELFNIVRK
VrBZR4       IINKFCQLID EGIRQELMHMKSLNVDGVVVDCWWGIVEGWSPQKYVWSGYRELFNIVRK
GmBZR6       IINKFCQLID EGIKQELIHIKSLNVDGVVVDCWWGIVEGWSSQKYVWSGYRELFNIIRE
GmBZR1       IINKFCQLID EGIKQELIHIKSLNVDGVVVDCWWGIVEGWSSQKYVWSGYRELFNIIRE
CcBZR6       IINKFCQLID EGIKQELIHIKTLNIDGVVVDCWWGIVEAWSSQKYVWSGYRELFNMIRE
```

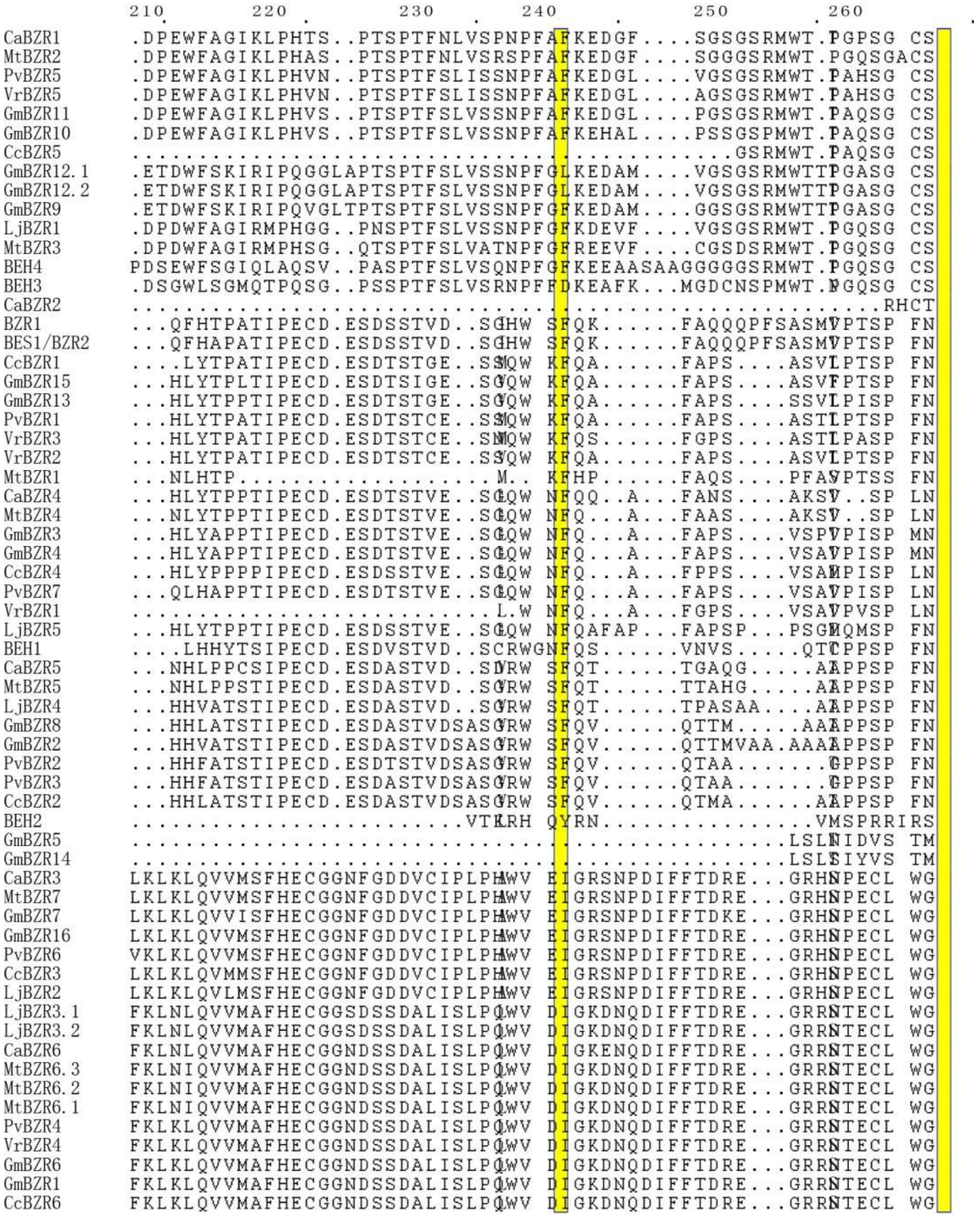
210 220 230 240 250 260
CaBZR1 .DPEWFAGIKLPHTS..PTSPTFNLVSPNPFAFKEDGF....SGSGSRMWT.PGPSG CS
MtBZR2 .DPEWFAGIKLPHAS..PTSPTFNLVSRSPFAFKEDGF....SGGGSRMWT.PGQSGACS
PvBZR5 .DPEWFAGIKLPHVN..PTSPTFSLISSNPFAFKEDGL....VGSGSRMWT.PAHSG CS
VrBZR5 .DPEWFAGIKLPHVN..PTSPTFSLISSNPFAFKEDGL....AGSGSRMWT.PAHSG CS
GmBZR11 .DPEWFAGIKLPHVS..PTSPTFSLVSSNPFAFKEDGL....PGSGSRMWT.PAQSG CS
GmBZR10 .DPEWFAGIKLPHVS..PTSPTFSLVSSNPFAFKEHAL....PSSGSPMWT.PAQSG CS
CcBZR5 ...........................................GSRMWT.PAQSG CS
GmBZR12.1 .ETDWFSKIRIPQGGLAPTSPTFSLVSSNPFGLKEDAM....VGSGSRMWTTPGASG CS
GmBZR12.2 .ETDWFSKIRIPQGGLAPTSPTFSLVSSNPFGLKEDAM....VGSGSRMWTTPGASG CS
GmBZR9 .ETDWFSKIRIPQVGLTPTSPTFSLVSSNPFGFKEDAM....GGSGSRMWTTPGASG CS
LjBZR1 .DPDWFAGIRMPHGG..PNSPTFSLVSSNPFGFKDEVF....VGSGSRMWT.PGQSG CS
MtBZR3 .DPDWFAGIRMPHSG..QTSPTFSLVATNPFGFREEVF....CGSDSRMWT.PGQSG CS
BEH4 PDSEWFSGIQLAQSV..PASPTFSLVSQNPFGFKEEAASAAGGGGGSRMWT.PGQSG CS
BEH3 .DSGWLSGMQTPQSG..PSSPTFSLVSRNPFFDKEAFK...MGDCNSPMWT.NGQSG CS
CaBZR2 .......................................................RHCT
BZR1 ...QFHTPATIPECD.ESDSSTVD..SGHW SFQK......FAQQQPFSASMVPTSP FN
BES1/BZR2 ...QFHAPATIPECD.ESDSSTVD..SGHW SFQK......FAQQQPFSASMVPTSP FN
CcBZR1 ....LYTPATIPECD.ESDTSTGE..SSMQW KFQA......FAPS....ASVLPTSP FN
GmBZR15 ...HLYTPLTIPECD.ESDTSIGE..SGYQW KFQA......FAPS....ASVFPTSP FN
GmBZR13 ...HLYTPPTIPECD.ESDTSTGE..SGYQW KFQA......FAPS....SSVLPISP FN
PvBZR1 ...HLYTPATIPECD.ESDTSTCE..SSMQW KFQA......FAPS....ASTLPTSP FN
VrBZR3 ...HLYTPATIPECD.ESDTSTCE..SNMQW KFQS......FGPS....ASTLPASP FN
VrBZR2 ...HLYTPATIPECD.ESDTSTCE..SSYQW KFQA......FAPS....ASVLPTSP FN
MtBZR1 ...NLHTP...................M.. KFHP......FAQS....PFASVPTSS FN
CaBZR4 ...HLYTPPTIPECD.ESDTSTVE..SGLQW NFQQ..A...FANS....AKSV..SP LN
MtBZR4 ...NLYTPPTIPECD.ESDTSTVE..SGLQW NFQ...A...FAAS....AKSV..SP LN
GmBZR3 ...HLYAPPTIPECD.ESDTSTVE..SGLQW NFQ...A...FAPS....VSPVPISP MN
GmBZR4 ...HLYAPPTIPECD.ESDTSTVE..SGLQW NFQ...A...FAPS....VSAVPISP MN
CcBZR4 ...HLYPPPPIPECD.ESDSSTVE..SGLQW NFQ...A...FPPS....VSAMPISP LN
PvBZR7 ...QLHAPPTIPECD.ESDTSTVE..SGLQW NFQ...A...FAPS....VSAVPISP LN
VrBZR1 ...........................L.W NFQ...A...FGPS....VSAVPVSP LN
LjBZR5 ...HLYTPPTIPECD.ESDSSTVE..SGLQW NFQAFAP...FAPSP...PSGMQMSP FN
BEH1 ....LHHYTSIPECD.ESDVSTVD..SCRWGNFQS......VNVS.....QTCPPSP FN
CaBZR5 ...NHLPPCSIPECD.ESDASTVD..SDRW SFQT......TGAQG.....AAPPSP FN
MtBZR5 ...NHLPPSTIPECD.ESDASTVD..SGRW SFQT......TTAHG.....AAPPSP FN
LjBZR4 ...HHVATSTIPECD.ESDASTVD..SGRW SFQT......TPASAA....AAPPSP FN
GmBZR8 ...HHLATSTIPECD.ESDASTVDSASGRW SFQV......QTTM.....AAAPPSP FN
GmBZR2 ...HHVATSTIPECD.ESDASTVDSASGRW SFQV......QTTMVAA.AAAAPPSP FN
PvBZR2 ...HHFATSTIPECD.ESDTSTVDSASGRW SFQV......QTAA.......GPPSP FN
PvBZR3 ...HHFATSTIPECD.ESDASTVDSASGRW SFQV......QTAA.......GPPSP FN
CcBZR2 ...HHLATSTIPECD.ESDASTVDSASGRW SFQV......QTMA......AAPPSP FN
BEH2 .........................VTKLRH QYRN................VMSPRRIRS
GmBZR5 ...................................................LSLNIDVS TM
GmBZR14 ...................................................LSLSIYVS TM
CaBZR3 LKLKLQVVMSFHECGGNFGDDVCIPLPHAWV EIGRSNPDIFFTDRE...GRHNPECL WG
MtBZR7 LKLKLQVVMSFHECGGNFGDDVCIPLPHAWV EIGRSNPDIFFTDRE...GRHNPECL WG
GmBZR7 LKLKLQVVISFHECGGNFGDDVCIPLPHAWV EIGRSNPDIFFTDKE...GRHNPECL WG
GmBZR16 LKLKLQVVMSFHECGGNFGDDVCIPLPHAWV EIGRSNPDIFFTDRE...GRHNPECL WG
PvBZR6 VKLKLQVVMSFHECGGNFGDDVCIPLPHAWV EIGRSNPDIFFTDRE...GRHNPECL WG
CcBZR3 LKLKLQVMMSFHECGGNFGDDVCIPLPHAWV EIGRSNPDIFFTDRE...GRHNPECL WG
LjBZR2 LKLKLQVLMSFHECGGNFGDDVCIPLPHAWV EIGRSNPDIFFTDRE...GRHNPECL WG
LjBZR3.1 FKLNLQVVMAFHECGGSDSSDALISLPQLWV DIGKDNQDIFFTDRE...GRRNTECL WG
LjBZR3.2 FKLNLQVVMAFHECGGSDSSDALISLPQLWV DIGKDNQDIFFTDRE...GRRNTECL WG
CaBZR6 FKLNLQVVMAFHECGGNDSSDALISLPQLWV DIGKENQDIFFTDRE...GRRNTECL WG
MtBZR6.3 FKLNIQVVMAFHECGGNDSSDALISLPQLWV DIGKDNQDIFFTDRE...GRRNTECL WG
MtBZR6.2 FKLNIQVVMAFHECGGNDSSDALISLPQLWV DIGKDNQDIFFTDRE...GRRNTECL WG
MtBZR6.1 FKLNIQVVMAFHECGGNDSSDALISLPQLWV DIGKDNQDIFFTDRE...GRRNTECL WG
PvBZR4 FKLKLQVVMAFHECGGNDSSDALISLPQLWV DIGKDNQDIFFTDRE...GRRNTECL WG
VrBZR4 FKLKLQVVMAFHECGGNDSSDALISLPQLWV DIGKDNQDIFFTDRE...GRRNTECL WG
GmBZR6 FKLKLQVVMAFHECGGNDSSDALISLPQLWV DIGKDNQDIFFTDRE...GRRNTECL WG
GmBZR1 FKLKLQVVMAFHECGGNDSSDALISLPQLWV DIGKDNQDIFFTDRE...GRRNTECL WG
CcBZR6 FKLKLQVVMAFHECGGNDSSDALISLPQLWV DIGKDNQDIFFTDRE...GRRNTECL WG

```
                                                     270       .     280      .   290          .
CaBZR1        PAIAAGS.................DHNADIPMSEAAEISD FAFGSNTF.....P.WGIVK
MtBZR2        PAIAAGF.................DQTADIPMSEA.EISD FAFGSNTF.....P.WGIVK
PvBZR5        PAIAPGT.................DHNADIPMSEA.EVSD FAFGSNML.....P.WGLVK
VrBZR5        PAVAAGT.................DQNADIPMSEA.EVSD FAFGSNML.....P.WGLVK
GmBZR11       PAIPPGS.................DQNADIPMSEA.EVSD FAFGSNTL.....P.WGLVK
GmBZR10       PAVPPGS.................YQNADIPMSDA.EVSD FAFGSNVL.....P.WGLVK
CcBZR5        PAVPPGS.................DHTADIPMSEA.EVSD FAFGSNML.....P.WGLVK
GmBZR12.1     PAVAAGS.................ENTSDIPMAEA.EVSD FAFGSSSS......WGLVNA
GmBZR12.2     PAVAAGS.................ENTSDIPMAEA.EVSD FAFGSSSS......WGLVNA
GmBZR9        PAVAAGS.................ENTSDIPMAEA.EVSD FAFGSSSS......WLVNA
LjBZR1        AAIAAGS.................DHTADIPMAEA.EVSD FAFGSSTA.....P.WGLVK
MtBZR3        PALAAGS.................DHNADIPMSEA.EISD FAFGSSAV.....P.WGLVK
BEH4          PAIPPGA.................DQTADVPMSEAVEAPP FAFGSNTN......WGLVKA
BEH3          PAIPAGV.................DQNSDVPMADG.EMTA FAFGCNAMAAN..P.WGMVK
CaBZR2        WHMAHAT.................DASDDS.............................
BZR1          LVKPAP................QQMSPNTAAFQEIGHQSS FKFENSQ......P.W..VK
BES1/BZR2     LVKPAP................QQLSPNTAAIQEIGHQSS FKFENSQ......P.W..VK
CcBZR1        LVKPMV................PHNVPDNSIQEMRTESSE FGVQ.........P.W..VK
GmBZR15       LVKPVI................PHRMPDNSIQVMRTESSE FGVQ.........P.W..VK
GmBZR13       LVKPVV................PPGMPDNSIQEMRTESSD FGVQ.........P.W..VK
PvBZR1        LVKPLV................PHIVPDNSIQEMRTESSE IGVQ.........P.W..VM
VrBZR3        LVKPLV................PHGVPDNSIQEMRTESSE FGVQ.........P.W..VK
VrBZR2        LVKPVV................PPGVPDNSIQEMRTESSE IGVQ.........P.W..VM
MtBZR1        .......................AVADNSFSEK.TSSKMLGVQ.........P.W..VK
CaBZR4        FMKPVIIEQQNN............LLPDNRIRELRIESEP FGVQV........P.W..VK
MtBZR4        FMKPVINEQHN.............MLPHNRMQEMRIESEP FGVQ.........P.W..VK
GmBZR3        FIKPVVSQQHKH..........NLNLPGNGIQEMRIESEP FAMQ.........P.W..VK
GmBZR4        FIKPVVSQQHKH..........NLNLSGNGIQEMRIESEP FAMQ.........P.W..VK
CcBZR4        FIKPVVNQQH..............NLP...IQEERIG.....VQ.........P.W..VK
PvBZR7        FIKPVVSQQHMH..........NLNHPDNRIQEMRNSEVQFGVQ.........P.W..VK
VrBZR1        FIKPVASQQHKH..........NLNHPDNRIQEMRNSEVQFGVQ.........P.W..VK
LjBZR5        FIKPVMRQQQ..............NMPDSRDQEMRSDSEA FGVQ.........P.W..VK
BEH1          LVG..........................KSVSSVGVDVS.........P.W..VK
CaBZR5        LMKPVMQKITPQGSMDMIHMNEGMQWASSSAAERGRDG.S FDFENGRV.....P.W..VK
MtBZR5        LMKPAMQ.ITPQSSMDMKHMNEAMQWSAGSATERGRDG.S FDFENGRV.....P.W..VK
LjBZR4        LVKPAIQMITPQSSMD...MNEGLPWGSG..AEIGRDG.S FDFENGR......P.W..VK
GmBZR8        LMKPAMQQIAAQ.........EGMLWGSV..AERVRDGGS FDFENGR......P.W..VK
GmBZR2        LMKPAMQQIAAQ.........EGMQWGSV..AERGRDGGS FDFENGR......P.W..VK
PvBZR2        LIKPAMHQIPAQ.........DAVQWGSV..AEIGIDGVS FDFENGR......P.W..VK
PvBZR3        LIKPAMHQIPAQ.........DAVQWGSV..AEIGIDGVS FDFENGR......P.W..VK
CcBZR2        LMKPAMQIGSQD.......VNDGMQMQWE......RDG.S FDFENGR......P.W..VK
BEH2          RIQ............................EGGSISNLLLL...............
GmBZR5        IITPGP....................VVDFLISNQNVRDPFSLD..........W.....
GmBZR14       IITPGP....................VVDFLISNQNVRDPFSLD..........W.....
CaBZR3        IDKERVLRGRTAVEVYFDFMRSFRVEFDQFFEDGFIESMI VGLGPCGELRYPSCWTVKH
MtBZR7        IDKERVLRGRTAVEVYFDFMRSFRIEFNEYFEDGFIESMI VGLGPCGELRYPSCWPVKH
GmBZR7        IDKERVLRGRTAVEVYFDFMRSFRVEFDEYFEDGFIESMI IGLGPCGELRYPSCWPVKH
GmBZR16       IDKERVLRGRTALEVYFDFMRSFRVEFDEYFEDGLIESMI VGLGPCGELRYPSCWPVKH
PvBZR6        IDKERVLRGRTAVEVYFDFMRSFRVEFDEYFEDGLIESMV VGLGPCGELRYPSCWPVKH
CcBZR3        IDKERVLRGRTAVEVYFDFMRSFRVEFDEYFEDGLIESMI VGLGPCGELRYPSSWPVKH
LjBZR2        IDKERVLRGRTAVEVYFDFMRSFRVEFDEYFEDGFIESMI VGLGPCGELRYPSCWPVKH
LjBZR3.1      IDKERVLKGRTGIEVYFDMMRSFRTEFDDLFAEGLIESSV IGLGASGELKYPSFWSERM
LjBZR3.2      IDKERVLKGRTGIEVYFDMMRSFRTEFDDLFAEGLIESSV IGLGASGELKYPSFWSERM
CaBZR6        IDKERVLKGRTGIEVYFDMMRSFRTEFDDLFAEGLIEYAV IGLGASGELKYPSFWSERM
MtBZR6.3      IDKERVLKGRTGIEVYFDMMRSFRTEFDDLFAEGMIEDAV IGLGASGELKYPSFWSERM
MtBZR6.2      IDKERVLKGRTGIEVYFDMMRSFRTEFDDLFAEGMIEDAV IGLGASGELKYPSFWSERM
MtBZR6.1      IDKERVLKGRTGIEVYFDMMRSFRTEFDDLFAEGMIEDAV IGLGASGELKYPSFWSERM
PvBZR4        IDKERVLKGRTGIEVYFDMMRSFRTEFDDLFAEGLIEYAV VGLGASGELKYPSFWSERM
VrBZR4        IDKERVLKGRTGIEVYFDMMRSFRTEFDDLFAEGLIESAV VGLGASGELKYPSFWSERM
GmBZR6        IDKERVLKGRTGIEVYFDMMRSFRTEFDDLFAEGLIESAV VGLGASGELKYPSFWSERM
GmBZR1        IDKERVLKGRTGIEVYFDMMRSFRTEFDDLFAEGLIESAV VGLGASGELKYPSFWSERM
CcBZR6        IDKERVLKGRTGIEVYFDMMRSFRTEFDDLFAEGLIESAV IGLGASGELKYPSFWSERM
```

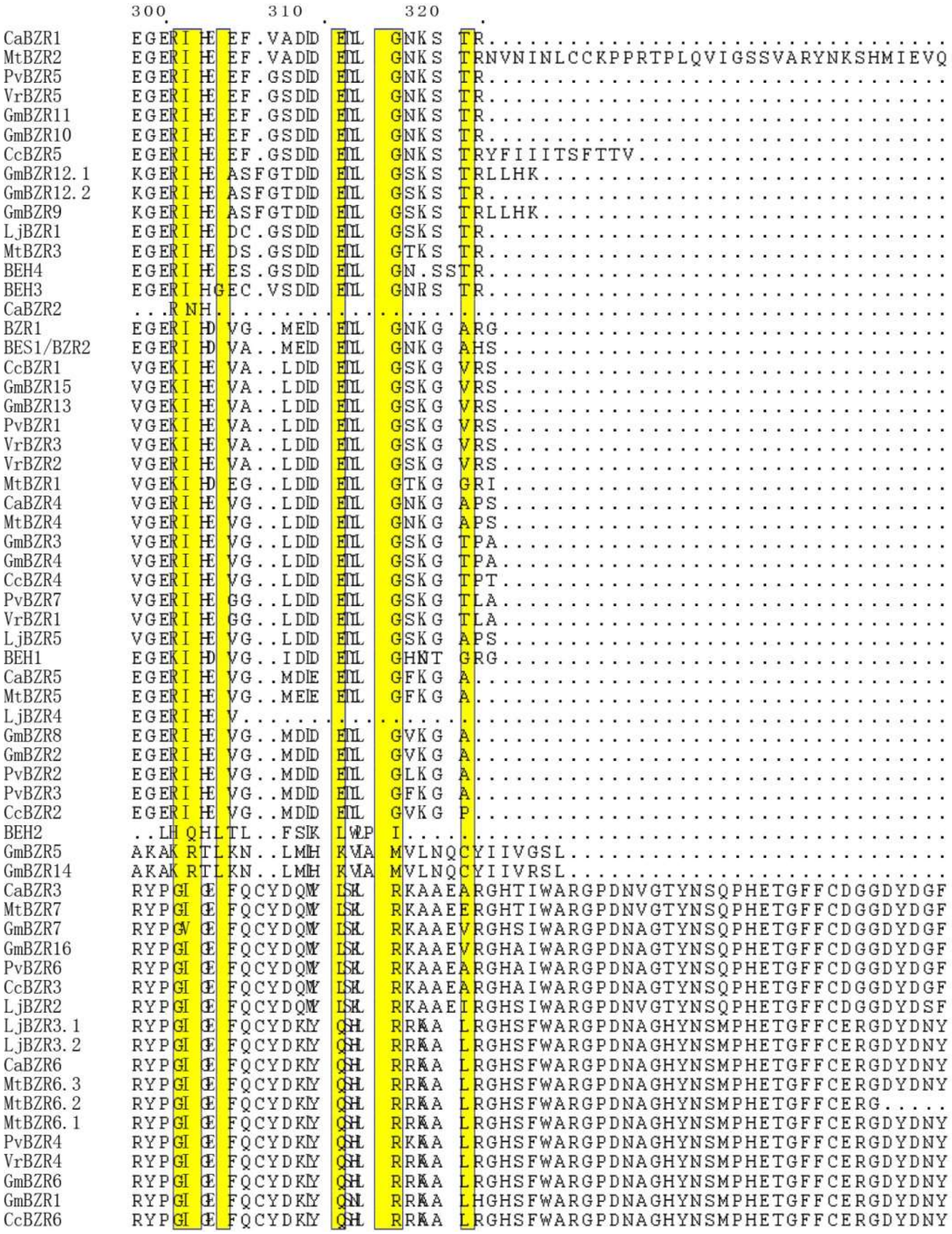

300 310 320
CaBZR1 EGERIHE EF.VADID EML GNKS TR..................................
MtBZR2 EGERIHE EF.VADID EML GNKS TRNVNINLCCKPPRTPLQVIGSSVARYNKSHMIEVQ
PvBZR5 EGERIHE EF.GSDID EML GNKS TR..................................
VrBZR5 EGERIHE EF.GSDID EML GNKS TR..................................
GmBZR11 EGERIHE EF.GSDID EML GNKS TR..................................
GmBZR10 EGERIHE EF.GSDID EML GNKS TR..................................
CcBZR5 EGERIHE EF.GSDID EML GNKS TRYFIIITSFTTV.......................
GmBZR12.1 KGERIHE ASFGTDID EML GSKS TRLLHK..............................
GmBZR12.2 KGERIHE ASFGTDID EML GSKS TR..................................
GmBZR9 KGERIHE ASFGTDID EML GSKS TRLLHK..............................
LjBZR1 EGERIHE DC.GSDID EML GSKS TR..................................
MtBZR3 EGERIHE DS.GSDID EML GTKS TR..................................
BEH4 EGERIHE ES.GSDID EML GN.SSTR..................................
BEH3 EGERIHGEC.VSDID EML GNRS TR..................................
CaBZR2 ...RNH...................................................
BZR1 EGERIHD VG..MEID EML GNKG ARG.................................
BES1/BZR2 EGERIHD VA..MEID EML GNKG AHS.................................
CcBZR1 VGEKIHE VA..LDID EML GSKG VRS.................................
GmBZR15 VGEKIHE VA..LDID EML GSKG VRS.................................
GmBZR13 VGEKIHE VA..LDID EML GSKG VRS.................................
PvBZR1 VGEKIHE VA..LDID EML GSKG VRS.................................
VrBZR3 VGEKIHE VA..LDID EML GSKG VRS.................................
VrBZR2 VGERIHE VA..LDID EML GSKG VRS.................................
MtBZR1 VGEKIHD EG..LDID EML GTKG GRI.................................
CaBZR4 VGERIHE VG..LDID EML GNKG APS.................................
MtBZR4 VGERIHE VG..LDID EML GNKG APS.................................
GmBZR3 VGERIHE VG..LDID EML GSKG TPA.................................
GmBZR4 VGERIHE VG..LDID EML GSKG TPA.................................
CcBZR4 VGERIHE VG..LDID EML GSKG TPT.................................
PvBZR7 VGERIHE GG..LDID EML GSKG TLA.................................
VrBZR1 VGERIHE GG..LDID EML GSKG TLA.................................
LjBZR5 VGERIHE VG..LDID EML GSKG APS.................................
BEH1 EGEKIHD VG..IDID EML GHNT GRG.................................
CaBZR5 EGERIHE VG..MDIE EML GFKG A...................................
MtBZR5 EGERIHE VG..MEIE EML GFKG A...................................
LjBZR4 EGERIHE V...................................................
GmBZR8 EGERIHE VG..MDID EML GVKG A...................................
GmBZR2 EGERIHE VG..MDID EML GVKG A...................................
PvBZR2 EGERIHE VG..MDID EML GLKG A...................................
PvBZR3 EGERIHE VG..MDID EML GFKG A...................................
CcBZR2 EGERIHE VG..MDID EML GVKG P...................................
BEH2 ..LHQHLTL..FSIK LWLP I.....................................
GmBZR5 AKAKRTLKN..LMIH KVIA MVLNQCYIIVGSL............................
GmBZR14 AKAKRTLKN..LMIH KVIA MVLNQCYIIVRSL............................
CaBZR3 RYPGICE FQCYDQMY LSKL RKAAEARGHTIWARGPDNVGTYNSQPHETGFFCDGGDYDGF
MtBZR7 RYPGICE FQCYDQMY LSKL RKAAEERGHTIWARGPDNVGTYNSQPHETGFFCDGGDYDGF
GmBZR7 RYPGVCE FQCYDQMY LSKL RKAAEVRGHSIWARGPDNAGTYNSQPHETGFFCDGGDYDGF
GmBZR16 RYPGICE FQCYDQMY LSKL RKAAEVRGHAIWARGPDNAGTYNSQPHETGFFCDGGDYDGF
PvBZR6 RYPGICE FQCYDQMY LSKL RKAAEARGHAIWARGPDNAGTYNSQPHETGFFCDGGDYDGF
CcBZR3 RYPGICE FQCYDQMY LSKL RKAAEARGHAIWARGPDNAGTYNSQPHETGFFCDGGDYDGF
LjBZR2 RYPGICE FQCYDQMY LSKL RKAAEIRGHSIWARGPDNVGTYNSQPHETGFFCDGGDYDSF
LjBZR3.1 RYPGICE FQCYDKIY QSHL RRKA LRGHSFWARGPDNAGHYNSMPHETGFFCERGDYDNY
LjBZR3.2 RYPGICE FQCYDKIY QSHL RRKA LRGHSFWARGPDNAGHYNSMPHETGFFCERGDYDNY
CaBZR6 RYPGICE FQCYDKIY QSHL RRKA LRGHSFWARGPDNAGHYNSMPHETGFFCERGDYDNY
MtBZR6.3 RYPGICE FQCYDKIY QSHL RRKA LRGHSFWARGPDNAGHYNSMPHETGFFCERGDYDNY
MtBZR6.2 RYPGICE FQCYDKIY QSHL RRKA LRGHSFWARGPDNAGHYNSMPHETGFFCERG.....
MtBZR6.1 RYPGICE FQCYDKIY QSHL RRKA LRGHSFWARGPDNAGHYNSMPHETGFFCERGDYDNY
PvBZR4 RYPGICE FQCYDKIY QSHL RKKA LRGHSFWARGPDNAGHYNSMPHETGFFCERGDYDNY
VrBZR4 RYPGICE FQCYDKIY QSHL RRKA LRGHSFWARGPDNAGHYNSMPHETGFFCERGDYDNY
GmBZR6 RYPGICE FQCYDKIY QSHL RRKA LRGHSFWARGPDNAGHYNSMPHETGFFCERGDYDNY
GmBZR1 RYPGICE FQCYDKIY QSNL RRKA LHGHSFWARGPDNAGHYNSMPHETGFFCERGDYDNY
CcBZR6 RYPGICE FQCYDKIY QSHL RRKA LRGHSFWARGPDNAGHYNSMPHETGFFCERGDYDNY

```
CaBZR1      ............................................................
MtBZR2      GLLRSKTKKKHKYNQETKGVIRQVKQMKVVFGLQEVLEVVNNGIEVLPTNSHIDGKNKDG
PvBZR5      ............................................................
VrBZR5      ............................................................
GmBZR11     ............................................................
GmBZR10     ............................................................
CcBZR5      ............................................................
GmBZR12.1   ............................................................
GmBZR12.2   ............................................................
GmBZR9      ............................................................
LjBZR1      ............................................................
MtBZR3      ............................................................
BEH4        ............................................................
BEH3        ............................................................
CaBZR2      ............................................................
BZR1        ............................................................
BES1/BZR2   ............................................................
CcBZR1      ............................................................
GmBZR15     ............................................................
GmBZR13     ............................................................
PvBZR1      ............................................................
VrBZR3      ............................................................
VrBZR2      ............................................................
MtBZR1      ............................................................
CaBZR4      ............................................................
MtBZR4      ............................................................
GmBZR3      ............................................................
GmBZR4      ............................................................
CcBZR4      ............................................................
PvBZR7      ............................................................
VrBZR1      ............................................................
LjBZR5      ............................................................
BEH1        ............................................................
CaBZR5      ............................................................
MtBZR5      ............................................................
LjBZR4      ............................................................
GmBZR8      ............................................................
GmBZR2      ............................................................
PvBZR2      ............................................................
PvBZR3      ............................................................
CcBZR2      ............................................................
BEH2        ............................................................
GmBZR5      ............................................................
GmBZR14     ............................................................
CaBZR3      YGRFFLNWYSQVLVDHGNRVLSLAKLAFEGSCIAAKLSGIYWWYKTASHAAELTAGYYNP
MtBZR7      YGRFFLNWYSQALVDHGNRVLSMAKLAFEGSCIAAKVSGIYWWYKTASHAAELTSGYYNP
GmBZR7      YGRFFLSWYSQVLVDHGNRVLSLAKLAFEGSCIAAKLSGIYWWYKTASHAAELTAGYYNP
GmBZR16     YGRFFLSWYSQVLIDHGNRVLSLAKLAFEGSCIAAKLSGIYWWYKTASHAAELTAGYYNP
PvBZR6      YGRFFLSWYSQVLVDHGNRVLSLAKLAFEGSCVTAKLSGIYWWYKTASHAAELTAGYYNP
CcBZR3      YGRFFLSWYSQVLVDHGNRVLSLAKLAFEGSCIAAKLSGIYWWYKTASHAAELTAGYYNP
LjBZR2      YGRFFLNWYSQVLVDHGNRVLSLAKLAFEGSCIAAKLSGIYWWYKTASHAAELTAGYYNP
LjBZR3.1    YGRFFLHWYSQTLVDHADNVLSLASLAFEETQIIVKVPAVYWWYKTPSHAAELTAGYHNP
LjBZR3.2    YGRFFLHWYSQTLVDHADNVLSLASLAFEETQIIVKVPAVYWWYKTPSHAAELTAGYHNP
CaBZR6      YGRFFLHWYSQTLTDHADNVLSLASLAFEETKIIVKVPAVYWWYKSPSHAAELTAGYHNP
MtBZR6.3    YGRFFLHWYSQTLVDHADNVLSLANLAFEGTKIIVKV...VFWYIS..............
MtBZR6.2    .....................SCRILVVQDS.............................
MtBZR6.1    YGRFFLHWYSQTLVDHADNVLSLANLAFEGTKIIVKVPAVYWWYKTPSHAAELTAGYHNP
PvBZR4      YGRFFLHWYSQTLIDHADNVLSLATLAFEETKIIVKVPAVYWWYKTPSHAAELTAGYHNP
VrBZR4      YGRFFLHWYSQTLIDHADNVLSLATLAFEETKIIVKVPAVYWWYKTPSHAAELTAGYHNP
GmBZR6      YGRFFLHWYSQTLIDHADNVLSLATLAFEETKITVKVPAVYWWYKTPSHAAELTAGYHNP
GmBZR1      YGRFFLHWYSQTLIDHADNVLSLATLAFEETKIIVKVPAVYWWYKTPSHAAELTAGYHNP
CcBZR6      YGRFFLHWYSKTLIDHADNVLSLATLAFEETKIIVKVPAVYWWYKTPSHAAELTAGYHNP
```

| | |
|---|---|
| CaBZR1 | ............................................................ |
| MtBZR2 | KTLFYIHKCVGNKVFEKIVDANTSMEVWDTLVKYDGGDAKVKKMDEKIAYYFSTLVTITN |
| PvBZR5 | ............................................................ |
| VrBZR5 | ............................................................ |
| GmBZR11 | ............................................................ |
| GmBZR10 | ............................................................ |
| CcBZR5 | ............................................................ |
| GmBZR12.1 | ............................................................ |
| GmBZR12.2 | ............................................................ |
| GmBZR9 | ............................................................ |
| LjBZR1 | ............................................................ |
| MtBZR3 | ............................................................ |
| BEH4 | ............................................................ |
| BEH3 | ............................................................ |
| CaBZR2 | ............................................................ |
| BZR1 | ............................................................ |
| BES1/BZR2 | ............................................................ |
| CcBZR1 | ............................................................ |
| GmBZR15 | ............................................................ |
| GmBZR13 | ............................................................ |
| PvBZR1 | ............................................................ |
| VrBZR3 | ............................................................ |
| VrBZR2 | ............................................................ |
| MtBZR1 | ............................................................ |
| CaBZR4 | ............................................................ |
| MtBZR4 | ............................................................ |
| GmBZR3 | ............................................................ |
| GmBZR4 | ............................................................ |
| CcBZR4 | ............................................................ |
| PvBZR7 | ............................................................ |
| VrBZR1 | ............................................................ |
| LjBZR5 | ............................................................ |
| BEH1 | ............................................................ |
| CaBZR5 | ............................................................ |
| MtBZR5 | ............................................................ |
| LjBZR4 | ............................................................ |
| GmBZR8 | ............................................................ |
| GmBZR2 | ............................................................ |
| PvBZR2 | ............................................................ |
| PvBZR3 | ............................................................ |
| CcBZR2 | ............................................................ |
| BEH2 | ............................................................ |
| GmBZR5 | ............................................................ |
| GmBZR14 | ............................................................ |
| CaBZR3 | CNRDGYAAIMEMLKRNGV.................................LNAGWDVGL |
| MtBZR7 | CNRDGYAAITAMLKRNGVNLNIACVDLHTFN.QHESFPEPFADPERLVWQVLNAGWDVGL |
| GmBZR7 | CNRDGYAAIMTMLKTIGVSLNIPCVDLHTFNQQHEGFPETFADPEGIVWQLLNAGWDVDL |
| GmBZR16 | CNRDGYAAIMTMLKTNGINLNIPCVDLHTLN.QHEGFPETFADPEGLVWQVLNAGWEVDL |
| PvBZR6 | CNRDGYAAILTMLKINGVSLNIPCVELHTLN.QHEGFPETFADPEGLVWQVLNAGWDVDL |
| CcBZR3 | CNRDGYAAIMTMLKKNGVSLNIPCVELHSGN.QHEGFPETFADPEGLVWQVLNAGWDVDL |
| LjBZR2 | CNRDGYASIMTMLKRNGVSLNIPCVDLQTLN.QHEGFPETFADPEGLVWQVLNAGWDVGL |
| LjBZR3.1 | TNQDGYSPVFEVLKKHAVTMKFVCLGFHLSS...QEANESLVDPEGLSWQVLNSAWDRGL |
| LjBZR3.2 | TNQDGYSPVFEVLKKHAVTMKFVCLGFHLSS...QEANESLVDPEGLSWQVLNSAWDRGL |
| CaBZR6 | TNQDGYSPVFEVLRKHAVTMKFVCLGFNLSS...QEANESLVDPEGLSWQALNSAWERGL |
| MtBZR6.3 | .....................FSCCGFIL............................... |
| MtBZR6.2 | ............................................................ |
| MtBZR6.1 | TNQDGYSPVFEVLKKHAVTMKFVCLGFNPSN...QEANESLVDPDGLSWQVLNSAWERGL |
| PvBZR4 | TNQDGYYPVFEVLRKHAVTMKFVCLGFHLSS...QEANESLIDPEGLSWQVLNSAWDRGL |
| VrBZR4 | TNQDGYFPVFEVLRKHAVTMKFVCLGFHLSS...QEANESLIDPEGLSWQVLNSAWDRGL |
| GmBZR6 | TNQDGYSPVFEVLRKHAVTMKFVCLGFHLSS...QEANESLIDPEGLSWQVLNSAWDRGL |
| GmBZR1 | TYQDGYSPVFEVLRKHAVTMKFVCLGFHLSS...QEAYEPLIDPEGLSWQVLNSAWDRGL |
| CcBZR6 | TNQDGYSPLFEVLRKHAVTMKFVCLGFHLSS...QEANESLIDPEGLSWQVLNSAWDRGL |

```
CaBZR1       ............................................................
MtBZR2       QMKQCSDIMTHQVEDIQGTLEAHEMKITKRGNERQEEQANFSKFKKD.............
PvBZR5       ............................................................
VrBZR5       ............................................................
GmBZR11      ............................................................
GmBZR10      ............................................................
CcBZR5       ............................................................
GmBZR12.1    ............................................................
GmBZR12.2    ............................................................
GmBZR9       ............................................................
LjBZR1       ............................................................
MtBZR3       ............................................................
BEH4         ............................................................
BEH3         ............................................................
CaBZR2       ............................................................
BZR1         ............................................................
BES1/BZR2    ............................................................
CcBZR1       ............................................................
GmBZR15      ............................................................
GmBZR13      ............................................................
PvBZR1       ............................................................
VrBZR3       ............................................................
VrBZR2       ............................................................
MtBZR1       ............................................................
CaBZR4       ............................................................
MtBZR4       ............................................................
GmBZR3       ............................................................
GmBZR4       ............................................................
CcBZR4       ............................................................
PvBZR7       ............................................................
VrBZR1       ............................................................
LjBZR5       ............................................................
BEH1         ............................................................
CaBZR5       ............................................................
MtBZR5       ............................................................
LjBZR4       ............................................................
GmBZR8       ............................................................
GmBZR2       ............................................................
PvBZR2       ............................................................
PvBZR3       ............................................................
CcBZR2       ............................................................
BEH2         ............................................................
GmBZR5       ............................................................
GmBZR14      ............................................................
CaBZR3       PVISENALPCLNRVSYNKILDNTKPVSDPDGRHFSSFTYLRLSSLLMERQNFTEFERFVK
MtBZR7       PVVSENALPCLNRVSYNKVLDNTKPINDPDGRHFSSFTYLRLSPLLMERPNFIEFERFVK
GmBZR7       PVTGQNGFPCLNRVGYNKVLDNAKPMNDPDGRLFSSFTYLRLSPLLMEQQNFVEFERFVK
GmBZR16      PVTSQNGFPCLNRVGYNKVLDNAKPMNDPDGRHFSSFTYLRLSSLLMERQNFIEFERFVK
PvBZR6       PVTSQNGFPCLNRVSYNKVLDHAKPMNDPDGRHFSSFTYLRLSPVLMERQNFIEFERFVK
CcBZR3       PVTSQNAFPCLNRVSYNKLLDNAKPMNDPDGRHFSSFTYLRLSPPLMERQNFIEFERFVK
LjBZR2       PVVSENALPCLNRVSYNKVLDNAKPMNDPDGRHFSSFAYPRLSPLLMERQNFIEFERFVK
LjBZR3.1     IAAGENALLCYDREGHKRLVEMAKPRNDPDRRHFSFFVYQQPS.LLQGNPCLSELDFFIK
LjBZR3.2     IAAGENALLCYDREGHKRLVEMAKPRNDPDRRHFSFFVYQQPS.LLQGNPCLSELDFFIK
CaBZR6       ITAGENALFGYDRERYKRLVEMAKPRNDPDHRHFSFFVYQQPS.LLQGNVCLSELDFFIK
MtBZR6.3     ............................................................
MtBZR6.2     ............................................................
MtBZR6.1     ITSGENAIFCYDRERYERLIEMAKPRNDPDHRHFSFFVYQQPS.LLQGNVCLSELDFFIK
PvBZR4       MAGGENALLCYDREGYKRLVDTAKPRNDPDHRHFSFFVYQQPS.LLQANVCLSELDFFVK
VrBZR4       MAGGENALLCYDREGYKRLVDTAKPRNDPDHRHFSFFVYQQPS.LLQANVCLSDLDFFVK
GmBZR6       MAAGENALLCYDREGYKKLVEIAKPRNDPDRRHFSFFVYQQPS.LLQTNVCWSELDFFVK
GmBZR1       MAAGENALLCYGREGYKRLVEMAKPRNDPDCRHFSFFVYQQPS.LLQANVCLSELDFFVK
CcBZR6       MAVGENALLCYDREGYKRLVEMAKPRNDPDRRHFSFFVYQQPS.LLQANVCLSELDFFIK
```

```
CaBZR1       ......................
MtBZR2       ......................
PvBZR5       ......................
VrBZR5       ......................
GmBZR11      ......................
GmBZR10      ......................
CcBZR5       ......................
GmBZR12.1    ......................
GmBZR12.2    ......................
GmBZR9       ......................
LjBZR1       ......................
MtBZR3       ......................
BEH4         ......................
BEH3         ......................
CaBZR2       ......................
BZR1         ......................
BES1/BZR2    ......................
CcBZR1       ......................
GmBZR15      ......................
GmBZR13      ......................
PvBZR1       ......................
VrBZR3       ......................
VrBZR2       ......................
MtBZR1       ......................
CaBZR4       ......................
MtBZR4       ......................
GmBZR3       ......................
GmBZR4       ......................
CcBZR4       ......................
PvBZR7       ......................
VrBZR1       ......................
LjBZR5       ......................
BEH1         ......................
CaBZR5       ......................
MtBZR5       ......................
LjBZR4       ......................
GmBZR8       ......................
GmBZR2       ......................
PvBZR2       ......................
PvBZR3       ......................
CcBZR2       ......................
BEH2         ......................
GmBZR5       ......................
GmBZR14      ......................
CaBZR3       RMH....GEAVLDLQL......
MtBZR7       RMH....GEAVLDLQV......
GmBZR7       RMH....GEAVLDLQV......
GmBZR16      RMH....GEAVLDLQV......
PvBZR6       RMH....GEAVLDLQV......
CcBZR3       RMH....GELMKKILNLSFPSY
LjBZR2       RMH....GEAVLDLQI......
LjBZR3.1     CMHAILAGEMTGDL........
LjBZR3.2     CMH....GEMTGDL........
CaBZR6       CMH....GEMTGDL........
MtBZR6.3     ......................
MtBZR6.2     ......................
MtBZR6.1     CMH....GEMTGNL........
PvBZR4       CMH....GEMTDP.........
VrBZR4       CMH....GEMTDL.........
GmBZR6       CMH....GEMTDL.........
GmBZR1       CMH....GEMSDL.........
CcBZR6       CMH....GEMTDL.........
```
