# Supplementary figures and images for: Genome-Wide Identification, Characterization, and Expression Profiling of the Legume BZR Transcription Factor Gene Family
Source: Front Plant Sci. 2018 Sep 19;9:1332. doi: 10.3389/fpls.2018.01332 (PMC6156370; doi:10.3389/fpls.2018.01332)

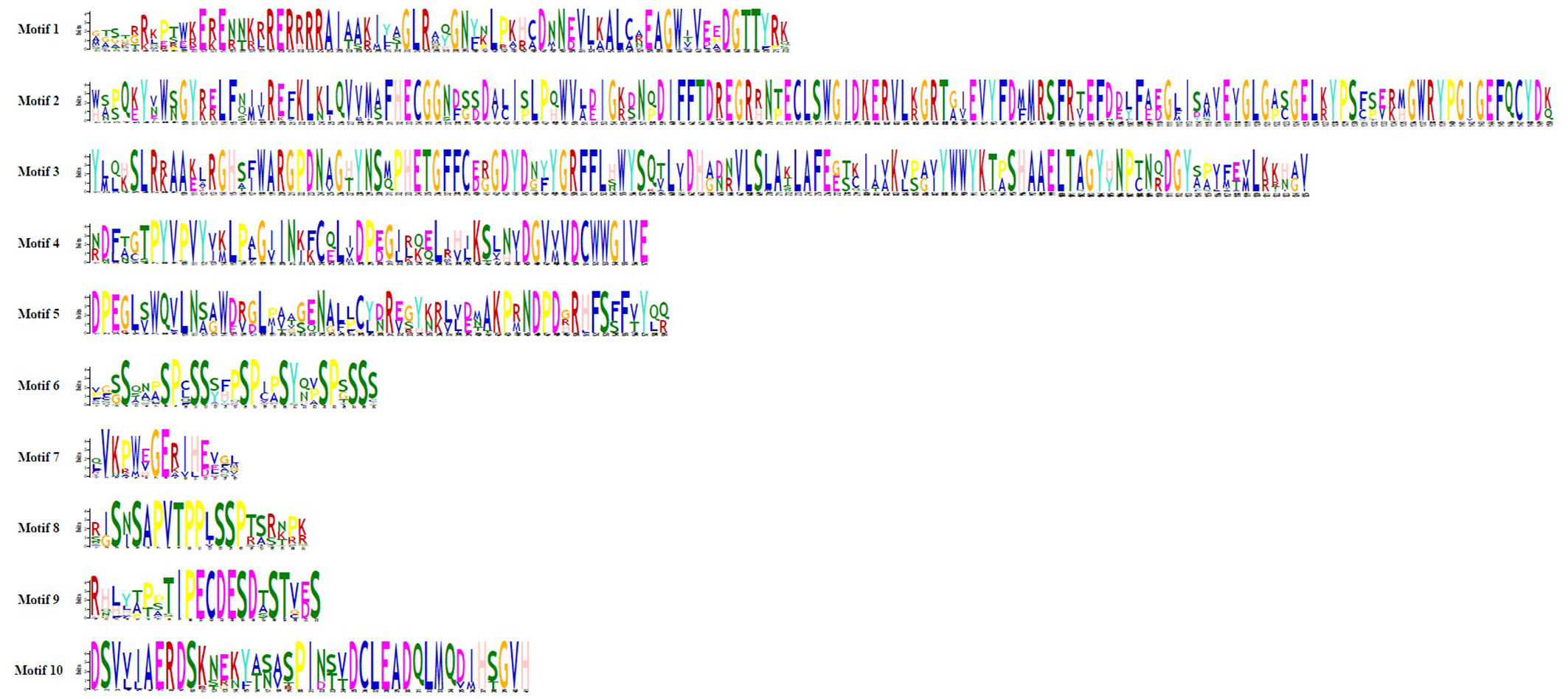

Supplement: FIGURE S1 — The conserved motifs of BZR genes. [file Image_1.JPEG]

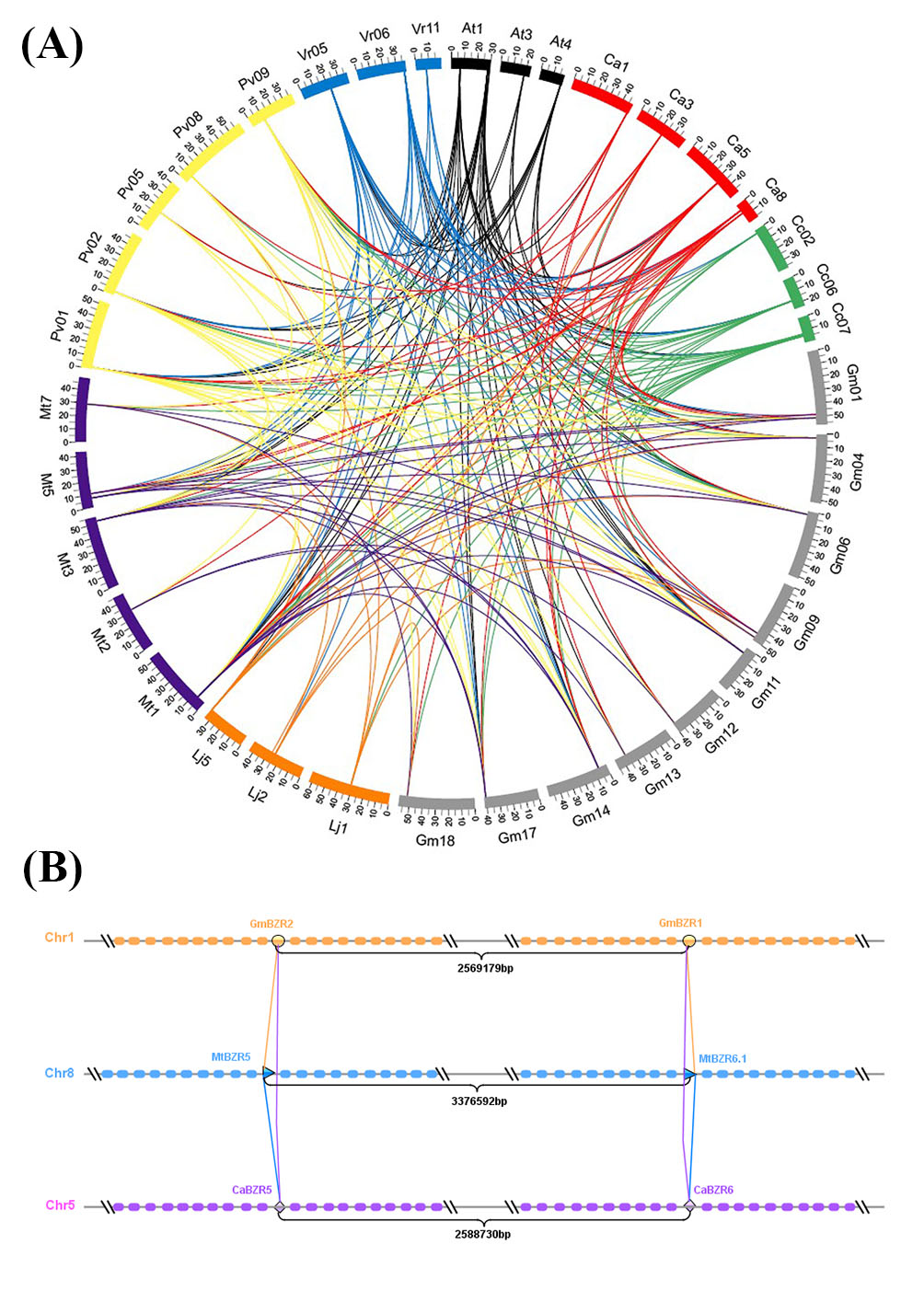

Supplement: FIGURE S2 — Synteny analysis of legume and Arabidopsis BZR genes. (A) Chromosomes from different species are depicted as different colored segments. The syntenic counterparts of conserved BZR genes between the genomes are interconnected by colored lines. (B) The micro- and macro-synteny relationship between GmBZR1/2 and their parallel syntenic blocks in other species. [file Image_2.JPEG]
